# Fate and Transformation of Landfill Leachate Dissolved Organic Nitrogen and Its Implications for Estuarine Algal Growth

**DOI:** 10.1021/acsestwater.6c00163

**Published:** 2026-05-18

**Authors:** Md Ashik Ahmed, Brian Brazil, Wenzheng Yu, Lifeng Zhang, Hans W. Paerl, Renzun Zhao

**Affiliations:** † Nanoengineering Department, Joint School of Nanoscience and Nanoengineering, 3616 North Carolina A&T State University, 2907 E Gate City Blvd, Greensboro, North Carolina 27401, United States; ‡ Waste Management Inc., Gaithersburg, Maryland 20878, United States; § State Key Laboratory of Environmental Aquatic Chemistry, Research Center for Eco-Environmental Sciences, Chinese Academy of Sciences, Beijing 100085, China; ∥ Department of Earth, Marine and Environmental Sciences, Institute of Marine Sciences, University of North Carolina Chapel Hill, Morehead City, North Carolina 28557, United States; ⊥ Civil, Architectural and Environmental Engineering Department, 3616 North Carolina A&T State University, Greensboro, North Carolina 27411, United States

**Keywords:** Algal bioassay, Biological nitrogen removal, Denitrification, Dissolved organic nitrogen, Estuarine eutrophication, Sewage, Landfill leachate

## Abstract

We investigated the fate and transformation of dissolved organic nitrogen (DON) during biological nitrogen removal (BNR) cotreatment of landfill leachate and municipal sewage. Three sequencing batch reactors (SBRs) were operated with high-, medium-, and no-leachate inputs. Systems were maintained at a solids retention time of 15 days and hydraulic retention time of 1 day, with methanol addition to support denitrification. Analytical methods included Fourier transform ion cyclotron resonance mass spectrometry (FTICR-MS), Fourier transform infrared spectroscopy (FTIR), carbon-13 nuclear magnetic resonance (^13^C NMR), 16S rRNA gene sequencing, and algal bioassays. All reactors achieved ∼99% ammonium removal and 87–92% total nitrogen removal, with effluent nitrate < 1.1 mg N/L. DON remained the dominant nitrogen form (73–80% of total nitrogen), with concentrations of 5.5, 6.5, and 2.2 mg N/L. FTICR-MS showed more bioavailable protein-like DON in high-leachate conditions (20%) and more refractory lignin-like DON in medium-leachate conditions (60%). Algal bioassays indicated limited short-term bioavailability. Results demonstrate that while BNR effectively removes dissolved inorganic nitrogen, it produces DON-rich effluent with composition-dependent ecological implications.

## Graphical abstract

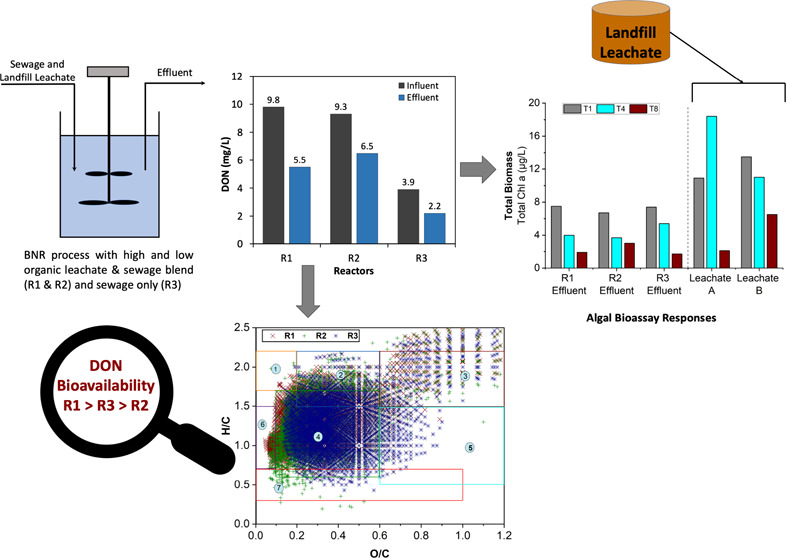

## 1 Introduction

Dissolved organic nitrogen (DON) is a potentially reactive nitrogen form that can influence algal growth and affect water quality in N-sensitive aquatic ecosystems.^1,2^ Despite advanced wastewater treatment processes, DON often persists in effluent, contributing to N loads in receiving waters.^3^ Globally, wastewater treatment plants (WWTPs) are incorporating BNR processes to meet stricter discharge standards, resulting in an increase in the proportion of DON from 10% to 80% of total dissolved N (TDN) in upgraded facilities, even as overall TDN levels in effluent decrease.^4,5^ This shift raises concerns, since most N removal methods target DIN, often neglecting biological processes producing DON in the effluent. While research on DON in sewage is well-documented, studies on landfill leachate as a source of DON still need to be expanded.

The BNR process effectively treats DIN by utilizing various microbial processes, such as nitrification-denitrification, short-cut nitrification, anammox, etc., while humic-derived N can pass through most BNR processes and contribute to the effluent total nitrogen. Nitrogen in landfill leachate requires a comprehensive assessment. Recent advances in landfill leachate treatment highlight the challenges of treating high-strength, carbon-limited leachate and the need for more robust nitrogen removal strategies.^6^ Previous studies showed that DON in landfill leachate exists mainly as protein-based or humic-derived N.^7^ Proteinaceous N can readily be converted to NH_4_^+^-N, whereas humic-derived N resists biodegradation. Accordingly, this study hypothesizes that differences in leachate organic composition produce protein-dominated or humic-dominated DON, which affects microbial transformation during BNR, while treated effluent DON is expected to show limited short-term bioavailability to algae regardless of the leachate source.

To better understand the molecular characteristics of DON during wastewater treatment, researchers have used FTICR-MS^8–10^ to provide new insights. Studies using FTICR-MS for sewage discovered that, with stricter wastewater treatment levels (TN < 6 mg/L), DON molecules exhibit higher reactivity and biodegradability, lower aromaticity, and greater thermodynamic favorability in aerobic environments.^11^ However, very few previous studies have utilized FTICR-MS for landfill leachate-induced DON. Other methods, such as ^13^C nuclear magnetic resonance (NMR) and Fourier transform infrared (FTIR) spectroscopy, can also contribute to the characterization of DON in complex water matrices.^12^

Few studies have examined the linkage between DON discharge and algal growth.^13,14^ For example, lab-based algal bioassays indicated that hydrophobic DON is less bioavailable to algae such as *Selenastrum capricornutum*,^15^ while an *in situ* algal bioassay also demonstrated limited bioavailability of hydrophobic DON to *Skeletonema costatum*.^16^ Despite the potential for eutrophication, systematic studies on *in situ* algal responses to DON from waste sources remain limited. Most *in situ* algal bioassays have focused on DON’s effect from municipal wastewater effluents and sediment fluxes on algal growth. For example, work conducted in Lake Taihu, China showed that DON released from sediments under sunlight can sustain algal blooms,^17^ while work in the Chesapeake Bay, USA, examined how wastewater-derived DON controls algal productivity.^18^ Existing studies rarely integrate DON molecular composition, microbial transformation, and *in situ* ecological response, leaving uncertainty about the fate and eutrophication risk of landfill leachate-derived DON after biological treatment. This study addresses that gap by examining DON fate and bioavailability during BNR cotreatment of landfill leachates with municipal sewage and by conducting the first in situ estuarine algal bioassay. By linking molecular, microbial, and ecological assessments, this work clarifies the environmental significance of leachate-driven effluent DONs beyond conventional DIN metrics.

## 2 Materials and Methods

### 2.1 Raw Sewage and Landfill Leachate

Raw sewage was collected from the Lexington Sewer Treatment Plant (LSTP) in NC, USA, at the influent station after primary screening every 15–20 days. The LSTP has a treatment capacity of 6.5 million gallons/day. Samples were stored at 4 °C in 5-gallon high-density polyethylene (HDPE) buckets and brought to 22 °C before use. Two landfill leachates, labeled Leachate A (L-A) and Leachate B (L-B), were also used; L-A has a very high organic carbon content, while L-B contains relatively lower organic carbon concentration. L-A and L-B were sourced from municipal solid waste landfills in Virginia, USA. The leachates were stored at 4 °C in 5-gallon HDPE buckets upon arrival and brought to 22 °C before use. Table 1 summarizes the characteristics of the sewage and landfill leachates.

**Table 1:** Average Sewage Characteristics over the Entire Study Period (∼175 days) and Characteristics of Landfill Leachates

**Parameters**	**Sewage** ^f^ **(R3, sewage** reactor)	**Leachate A (R1**, **high organic leachate** **reactor** **)**	**Leachate B (R2**, **medium organic leachate** **reactor** **)**
**pH**	7.1 ± 0.1	5.8	7.9
**Total Nitrogen (TN)** (mgN/L)	36 ± 3	2,430	1,900
**TKN** ^a^ (mgN/L)	35 ± 3	2,350	1,860
**NO**_**3**_^**–**^ **-N** **(mg/L)**	0.35 ± 0.30	78	37.6
**NO**_**2**_^**–**^ **-N** **(mg/L)**	0.06 ± 0.02	1.9	0.86
**NH**_**4**_^**+**^ **-N** **(mg/L)**	31.1 ± 3.0	1,500	1,630
**COD** **(mg/L)**	336 ± 65	108,000	17,000
**BOD** ^b^ (mg/L)	192 ± 18	26,100	4,150
**Phosphorus** **(mg/L PO**_**4**_^**3‑**^ **)**	26.3 ± 4.0	534	470
**Alkalinity** **(mg/L CaCO**_**3**_ **)**	171 ± 10	7,630	3,990
**TS** ^c^ (mg/L)	N/A	113,000	18,900
**TSS** ^d^ (mg/L)	N/A	2,690	595
**TDS** ^e^ (mg/L)	N/A	110,000	18,300

a TKN = total Khjeldahl nitrogen.

b BOD = biochemical oxygen demand.

c The results of this study are shown in Figure 1. TS = total solids.

d The results of this study are shown in Figure 1. TSS = total suspended solids.

e TDS = total dissolved solid; N/A = not available.

f Average of Ten Sewage Samples.

### 2.2 BNR Process Setup

Three SBR reactors were used to facilitate BNR: R1 fed with L-A (very high organic) and sewage blend, R2 with L-B (relatively medium organic) and sewage blend, and R3 with exclusively sewage. Figure 1 shows the lab-scale SBR sketch and setup.

**Figure 1:**
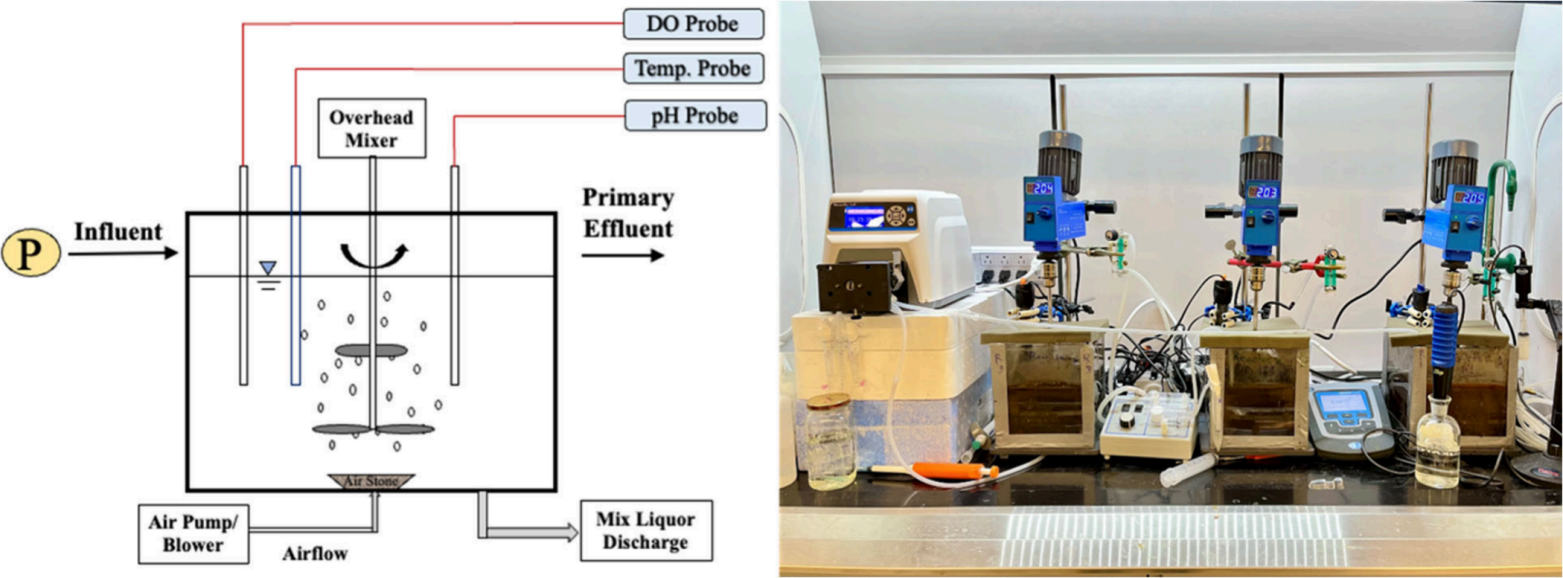
Schematic diagram (left) and the laboratory setup (right) of the lab-scale SBR reactors.

The reactors had 15 days of solid retention time (SRT) and 1 day of hydraulic retention time (HRT), with two cycles per day (12 h per cycle). The operation sequence of the 12-h cycle was aerobic (6.5 h), anoxic (3.5 h), postaerobic (1 h), settling, decanting-feeding, and idle (1 h). The aerobic phase’s dissolved oxygen (DO) level was maintained between 1 and 2 mg/L. R1 (high organic leachate reactor) and R2 (medium organic leachate reactor) maintained 4000 mg/L mixed liquor-suspended solids (MLSS), and R3 (sewage reactor) was 3000 mg/L. Initially, the reactors were fed sewage, and after attaining almost complete nitrification, R1 and R2 initiated leachate feeding with high organic (L-A) and medium organic leachate (L-B), respectively. R1, R2, and R3 received 0.77, 0.87, and 0.48 L/m^3^ of methanol, respectively, for optimal denitrification. Methanol was applied consistently across all reactors to ensure complete denitrification, allowing comparative evaluation of leachate effects independent of external carbon limitation. Leachate samples L-A (high organic leachate) and L-B (medium organic leachate) were added to R1 and R2 at 0.8% and 3.0% of influent volume. The leachate addition rates were selected based on target influent DON concentrations (∼10 mg/L) and reactor performance considerations. Prior studies have reported cotreatment ratios ranging from 0.02% to 10% (v/v), with system performance strongly dependent on leachate characteristics.^19–26^ A stepwise loading approach was employed, beginning at 0.2% and gradually increasing the leachate fraction to identify the maximum allowable loading without impairing nitrification. R1 exhibited nitrification inhibition beyond 0.8%, whereas R2 maintained stable performance up to 3%, indicating that these values represent operational thresholds rather than trivial contributions. This approach reflects realistic treatment conditions where leachate dosing is limited by process stability. The percentage nitrification was evaluated at each loading condition to determine the optimum ratio, as described in our previous study, which forms part of this work.^27^ No leachate was added to R3 during the study. Detailed descriptions are provided in the Supporting Information (SI) Section 1 and our previous study.^27^

### 2.3 Water Quality Analysis

The physicochemical analyses included pH, temperature, TN, NH_4_^+^-N, NO_2_^–^-N, NO_3_^–^-N, TKN, PO_4_^3–^, total alkalinity, COD, and BOD. pH was measured with the Apera Instruments AI311 Premium Series PH60 tester kit (Columbus, Ohio, USA), and DO was measured with a HACH Intellical LDO101 probe and HQ440 multimeter (Loveland, Colorado, USA). All other parameters were determined using the HACH DR6000 spectrophotometer with HACH TNT 822, 826, 830, 831, 832, 835, 839, 846, and 870 kits. DON was calculated by subtracting influent NH_4_^+^-N from influent TKN and effluent NH_4_^+^-N, NO_3_^–^-N, and NO_2_^–^-N from effluent TN.^7^ The American Public Health Association 5210 BOD test method was utilized for BOD testing.^28^ All the samples were filtered through 0.45 μm glass microfiber filter (GF/F) before analysis. Each analytical procedure was replicated three times for all samples to ensure the statistical validity of the results.

### 2.4 DON Molecular Characterization

FTICR-MS, FTIR, and ^13^C NMR were performed to investigate the molecular composition of DON.

A custom-built hybrid linear ion trap 21T FTICR-MS was used to analyze DON.^29^ Peaks above six times the baseline root-mean-square (RMS) noise at *m*/*z* 500 were exported to peak lists, and PetroOrg© software^30^ was used for molecular formula assignments and data visualization. The Van Krevelen diagram was plotted using molecular formulas. Further details of the FTICR-MS analysis method can be found in our group’s previous study.^31^

The samples were freeze-dried and then analyzed using an Attenuated Total Reflectance (ATR) Agilent 670 FTIR Spectrometer (Santa Clara, CA, USA). Each sample was applied to a diamond ATR crystal and recorded a unique 4000–550 cm^–1^ spectrum with 64 averaged scans in transmission mode with an 8 cm^–1^ resolution for accuracy.

Freeze-dried samples were also analyzed with an Agilent 400 MHz NMR (Santa Clara, CA, USA). For each sample, 50 mg was dissolved in 1 mL of deuterium oxide (D_2_O). Spectra were recorded over 24 h to improve ^13^C resolution, with water suppression at 4.66 ppm aiding peak clarity. Each spectrum was acquired using 115,000 scans with a 1-s acquisition, 1-s recycle, and a 45° pulse. Data were processed using 30 Hz line broadening and baseline correction prior to analysis. The details of the FTIR and NMR analysis methods used in this study have been described in our previous work.^27^

### 2.5 Algal Bioassay

The *in situ* algal bioassay was conducted using 0.01 N HCL and deionized water-washed polyethylene Cubitainers^R^ (Hedwin Corp., Newark, DE) as incubation vessels. Cubitainers are 85% transparent to photosynthetically active radiation (PAR) and chemically inert, suspended in a pond outside the UNC-IMS facility to mimic the natural light and temperature conditions (Figure S1).^32,33^ A layer of neutral density screening was placed over the Cubitainers, reducing incident light by 20% to avoid photoinhibition. The mixture of effluent samples and 200 μm filtered Neuse River (NR) water, NC estuarine surface water, was used for the experiment. The bioassay was designed to assess short-term algal response to effluent-derived DON under environmentally relevant conditions. Prior removal of inorganic nitrogen ensured that observed algal growth was primarily associated with DON bioavailability rather than DIN. Treatments included NR water, effluent, and nutrient spikes in triplicate, with thorough homogeneous mixing. The 11-day bioassay (T0 to T8 sampling time points) tested algal growth based on high-performance liquid chromatography (HPLC) analysis of photopigments diagnostic of major algal groups on GF/F (0.7 μm porosity) filtered water samples. All samples were analyzed in triplicate to ensure statistical validity. The algal bioassay and HPLC analysis methods used in this study have been described in detail in our previous work.^27^

### 2.6 Microbial Analysis

Activated sludge samples collected from R1 (high organic leachate reactor), R2 (medium organic leachate reactor), and R3 (sewage reactor) were taken in sterile 100 mL HDPE bottles, frozen, and sent to Microbial Insight Inc. (Knoxville, TN) for 16S rRNA next-generation sequencing. DNA extraction, sequencing with Illumina MiSeq-compatible primers, and quantitative polymerase chain reaction (qPCR) were performed on a QuantStudio 12K Flex Real-Time PCR System (Applied Biosystems, Grand Island, NY). The analysis was conducted in triplicate with an activated sludge sample. Microbial analysis method details are provided in SI section 4.

### 2.7 Statistical Analysis

DON data were analyzed in Minitab 17, using a two-sample *t* test and Anderson-Darling normality test. DON values were compared between R1 (high organic leachate reactor) and R2 (medium organic leachate reactor), between R1 (high organic leachate reactor) and R3 (sewage reactor), and between R2 (medium organic leachate reactor) and R3 (sewage reactor). All reported results are presented as the mean ± standard deviation, calculated from the number of samples analyzed, including three replicates, over the study period.

## 3 Results and Discussion

To assess the ecological relevance of landfill leachate–derived DON following biological treatment, the results are discussed along a treatment-to-ecosystem pathway. Reactor performance and DON persistence were first evaluated, followed by an examination of DON molecular composition and microbial community structure to elucidate transformation mechanisms. Finally, short-term algal responses to the treated effluents were assessed.

### 3.1 Sequencing Batch Reactors Performance

The pH levels remained stable throughout the study period for R1 (high organic leachate reactor), R2 (mediumorganic leachate reactor), and R3 (sewage reactor), averaging 7.7 ± 0.4, 7.6 ± 0.4, and 7.5 ± 0.5, respectively (Figure S2). The effluent COD (Figure 2(a), Table 2, Figures S3–S4) values for R1 (high organic leachate reactor), R2 (medium organic leachate reactor), and R3 (sewage reactor) were 279 ± 110 mg/L, 258 ± 80 mg/L, and 91 ± 40 mg/L, respectively, while the BOD (Figure 2(b), Table 2, Figures S5–S6) values were 86 ± 35 mg/L, 73 ± 40 mg/L, and 41 ± 10 mg/L, with removal efficiencies of 75%, 59%, and 72% for COD, and 79%, 77%, and 78% for BOD, respectively. Among the three reactors, the higher COD and BOD removal in the high organic leachate reactor (R1) is attributable to the greater fraction of bioavailable, readily degradable organic matter in leachate A. In contrast, the lower COD and BOD removal observed in the medium organic leachate reactor reflects the dominance of more refractory organic compounds in leachate B that resist microbial degradation. The sewage-fed reactor (R3) exhibited intermediate COD and BOD removal, indicating an organic matter bioavailability between that of high-organic leachate A and medium-organic leachate B. A comparable study used a lab-scale SBR for cotreatment of old landfill leachate and municipal wastewater and also reported COD removal efficiencies ranging from 60 to 73%, with maximum performance observed at 10% leachate concentration and 6-day HRT.^34^ In addition, we added excess external carbon (methanol) to promote complete denitrification contributed to elevated residual COD in the effluent, particularly in R3 (sewage reactor), where the added carbon was not fully oxidized during treatment.

**Table 2:** Influent and Primarily Treated Effluent Characteristics of the Sewage and Landfill Leachate Samples under Steady-State Conditions^b^

**Parameters**	**R1 Influent (high organic leachate reactor)** (99.2% sewage and 0.8% L-A)	**R1 Effluent (high organic leachate** reactor)	**R2 Influent (medium organic leachate reactor)** (97% sewage and 3% L-B)	**R2 Effluent (medium organic leachate** reactor)	**R3 Influent (sewage reactor)** (sewage only)	**R3 Effluent (sewage** reactor)
**pH**	7.1 ± 0.2	7.7 ± 0.4	7.2 ± 0.1	7.6 ± 0.4	7.1 ± 0.1	7.5 ± 0.5
**TN** (mg N/L)	56 ± 6	7 ± 2	91 ± 5	8 ± 2	36 ± 4	3 ± 1
**TKN** (mg N/L)	47 ± 2	6 ± 1	81 ± 2	8 ± 3	35 ± 2	3 ± 2
**NH**_**4**_^**+**^ **-N** (mg N/L)	45 ± 2	1 ± 1	81 ± 2	0.7 ± 0.3	31 ± 1	0.4 ± 0.2
**NO_3_^–^-N** (mg N/L)	0.9 ± 0.3	31 ± 3 *(postnitr.)* ^a^	0.4 ± 0.3	44 ± 2 *(postnitr.)* ^a^	0.5 ± 0.2	13 ± 2 *(postnitr.)* ^a^
	—	0.7 ± 0.2	—	1.1 ± 0.2	—	0.4 ± 0.1
**NO_2_^–^-N** (mg N/L)	0.09 ± 0.01	0.003 ± 0.002	0.07 ± 0.005	0.01 ± 0.003	0.06 ± 0.01	0.002 ± 0.002
**DON** (mg N/L)	10 ± 1	6 ± 1	9 ± 1	7 ± 1	4 ± 1	2 ± 1
**COD** (mg/L)	1140 ± 40	279 ± 110	640 ± 20	258 ± 80	320 ± 20	91 ± 40
**BOD** (mg/L)	410 ± 50	86 ± 40	310 ± 30	73 ± 40	180 ± 20	41 ± 10
**Alkalinity** **(mg/L as CaCO**_**3**_ **)**	200 ± 20	35 ± 4	220 ± 20	32 ± 8	200 ± 10	60 ± 20
**Phosphorus** **(mg P/L)**	32 ± 3	17 ± 3	39 ± 7	14 ± 2	27 ± 4	18 ± 2

a After the aerobic phase when nitrification is completed.

b The mean and standard deviation were calculated based on ∼100 influent and effluent samples (from Day 60 to 175 of the study period), each treatment in triplicate.

**Figure 2:**
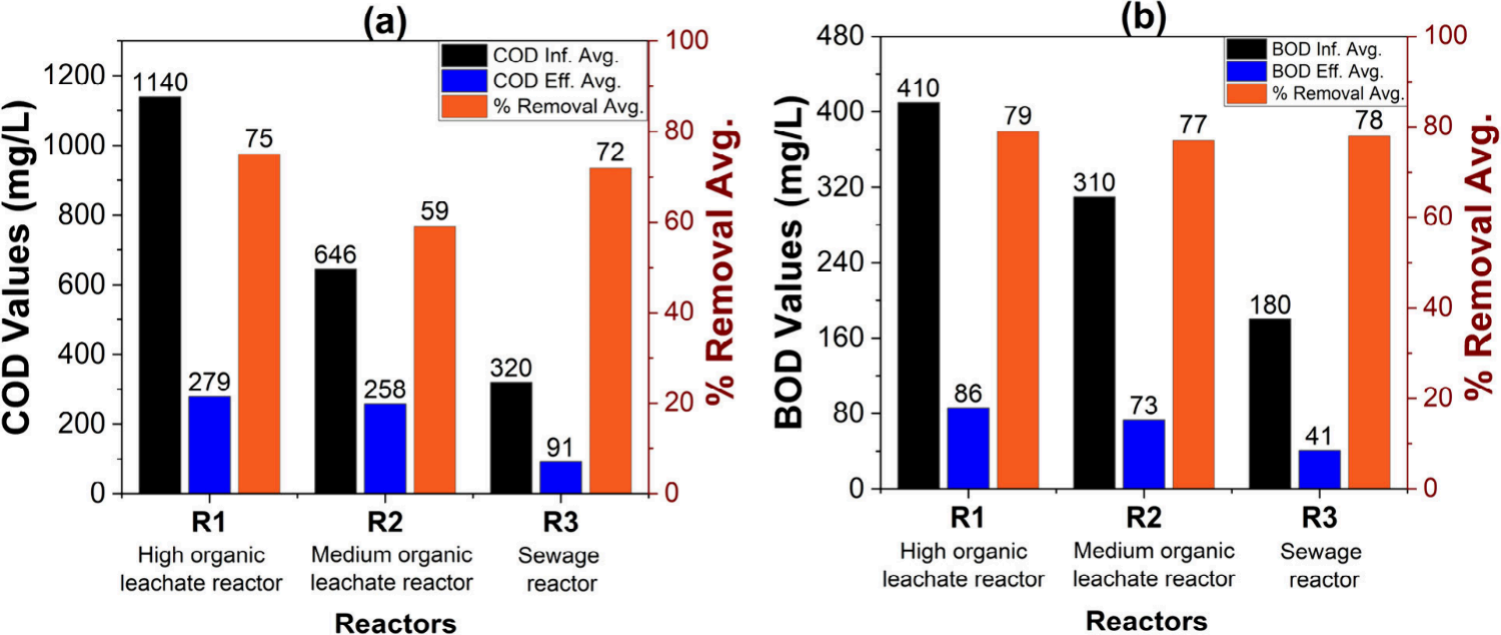
Average influent and effluent COD (a) and BOD (b) values with respective removal efficiency for R1 (high organic leachate reactor), R2 (meidum organic leachate reactor), and R3 (sewage reactor).

Effluent BOD concentrations in R1 (high organic leachate reactor) and R2 (medium organic leachate reactor) were more than the EPA discharge limit (≤30 mg/L),^35^ while R3 (sewage reactor) was slightly elevated. This outcome reflects the deliberate operational strategy to maximize denitrification and minimize residual DIN prior to downstream algal bioassays. To isolate short-term ecological effects of DON, DIN species (NH_4_^+^-N, NO_3_^–^-N, and NO_2_^–^-N) were minimized so that DON dominated the dissolved nitrogen pool, thereby avoiding DIN-driven algal responses.

BNR was highly effective across all reactors, resulting in a substantial reduction of total and inorganic nitrogen species. Total nitrogen (TN) concentrations decreased substantially from influent to effluent in all reactors, with removal efficiencies of 87%, 91%, and 92% for R1 (high organic leachate reactor), R2 (medium organic leachate reactor), and R3 (sewage reactor), respectively (Table 2). Influent TN concentrations of 55.6 ± 5.5, 90.7 ± 4.6, and 35.6 ± 4.1 mg/L were reduced to 6.8 ± 1.9, 8.2 ± 1.5, and 3.0 ± 0.9 mg/L in the effluents of R1 (high organic leachate reactor), R2 (medium organic leachate reactor), and R3 (sewage reactor), respectively. These TN removal efficiencies are consistent with a previous (AO)_2_ SBR study reporting average removals of approximately 88% under stable operation.^36^ In contrast, lower TN removal (63–66%) has been reported for SBRs treating synthetic wastewater with methanol or ethanol as the sole carbon source,^37^ highlighting the benefit of sewage–leachate cotreatment in providing a more diverse and bioavailable carbon pool under optimized operating conditions.

Effluent NH_4_^+^-N concentrations were consistently low (0.4–0.7 mg/L across all reactors), corresponding to removal efficiencies exceeding 98% (Figures 3 and S7) and confirming effective nitrification. These values are comparable to those reported by Hu et al. (2020), who observed NH_4_^+^-N removal efficiencies exceeding 94% in a sewage-based BNR system.^8^ Concurrent reductions in total Kjeldahl nitrogen (TKN) of 86.9–89.9% further indicate extensive nitrogen transformation. The accumulation of NO_3_^–^-N following nitrification (30.8 ± 3.4, 44.2 ± 2.2, and 12.7 ± 1.9 mg/L for R1 (high organic leachate reactor), R2 (medium organic leachate reactor), and R3 (sewage reactor), respectively) confirms complete ammonia oxidation with minimal residual NH_4_^+^-N in the effluents. Short-term fluctuations in nitrification observed between days 105 and 120 were attributed to operational interruption when reactors were fed once per day. As a result of effective DIN removal, the treated effluents were dominated by DON, providing a suitable basis for evaluating DON persistence, molecular composition, microbial transformation, and short-term ecological responses in subsequent sections.

**Figure 3:**
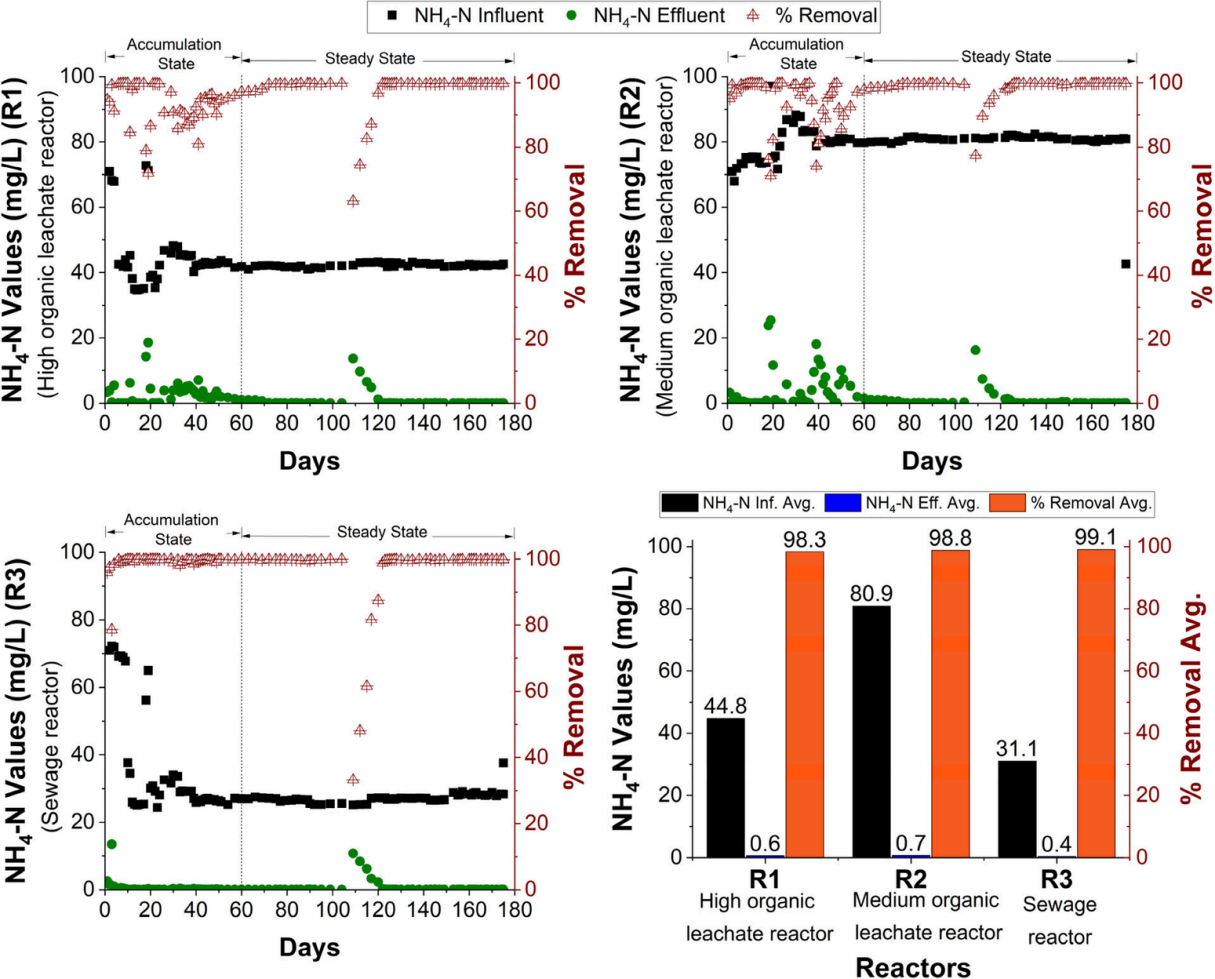
Influent and effluent NH_4_^+^-N values over the study period for R1 (high organic leachate reactor), R2 (medium organic leachate reactor), and R3 (sewage reactor) reactors with respective removal efficiency.

The addition of methanol significantly enhanced denitrification performance (P-value = 0.006; see SI Section 11), resulting in low effluent NO_3_^–^-N concentrations of 0.73 ± 0.20, 1.06 ± 0.15, and 0.42 ± 0.12 mg/L for R1 (high organic leachate reactor), R2 (medium organic leachate reactor), and R3 (sewage reactor), respectively (Table 2 and Figures S8–S9). During the initial 12 days of operation without methanol supplementation, elevated effluent NO_3_^–^-N concentrations were observed, indicating incomplete denitrification. Similar improvements in SBR denitrification efficiency with methanol addition have been reported previously.^38^ Effluent NO_2_^–^-N concentrations remained negligible across all reactors, indicating stable denitrification without nitrite accumulation. This ensured minimal interference from inorganic nitrogen during subsequent evaluation of effluent DON characteristics.

### 3.2 Effluent DON

Following effective removal of DIN (Section 3.1), DON became the dominant nitrogen fraction in the effluents, allowing for focused evaluation of its removal behavior and persistence during BNR treatment. Under steady-state conditions, the DON concentrations decreased from 9.8 ± 1.0 mg/L to 5.5 ± 1.2 mg/L (R1, high organic leachate reactor), from 9.3 ± 1.1 mg/L to 6.5 ± 1.1 mg/L (R2, medium organic leachate reactor), and from 3.9 ± 0.6 mg/L to 2.2 ± 0.5 mg/L (R3, sewage reactor) (Table 2, Figure 4, Figure S10), corresponding to removal efficiencies of 44.1%, 30.2%, and 43.8%, respectively. A two-sample *t* test indicated significant differences (*p* < 0.05) in effluent DON concentrations and removal efficiencies among the reactors. DON accounted for approximately 73–80% of TN in the effluents from all reactors, confirming its dominance following DIN removal. This shift in nitrogen speciation motivated further molecular-level investigation of effluent DON quality. To date, limited studies have explicitly evaluated DON removal during biological treatment of landfill leachate, restricting direct performance comparison. Reported effluent DON concentrations in BNR systems typically range from ∼0.76 to 10.9 mg N/L, accounting for ∼30–80% of total nitrogen, which is consistent with the DON levels (2.2–6.5 mg N/L; 73–80% of TN) observed in this study.^8,9,39–42^ R1 (0.8% high organic leachate) and R2 (3% medium organic leachate) exhibited comparable influent DON concentrations; however, R2 (medium organic leachate reactor) had substantially higher influent TN due to greater contributions from DIN. Despite similar influent DON levels, R1 (high organic leachate reactor) achieved greater DON removal than R2 (medium organic leachate reactor), suggesting that DON bioavailability, rather than concentration alone, governs removal efficiency during BNR treatment. The sewage-fed reactor (R3) exhibited DON removal efficiency comparable to R1 (hugh organic leachate reactor), indicating that sewage-derived DON shares similar biodegradability characteristics with DON in high-organic leachate. In contrast, the lower DON removal observed in R2 (medium organic leachate reactor) reflects the more refractory nature of DON associated with medium-organic leachate, which limits microbial degradation within the reactor.^8^

**Figure 4:**
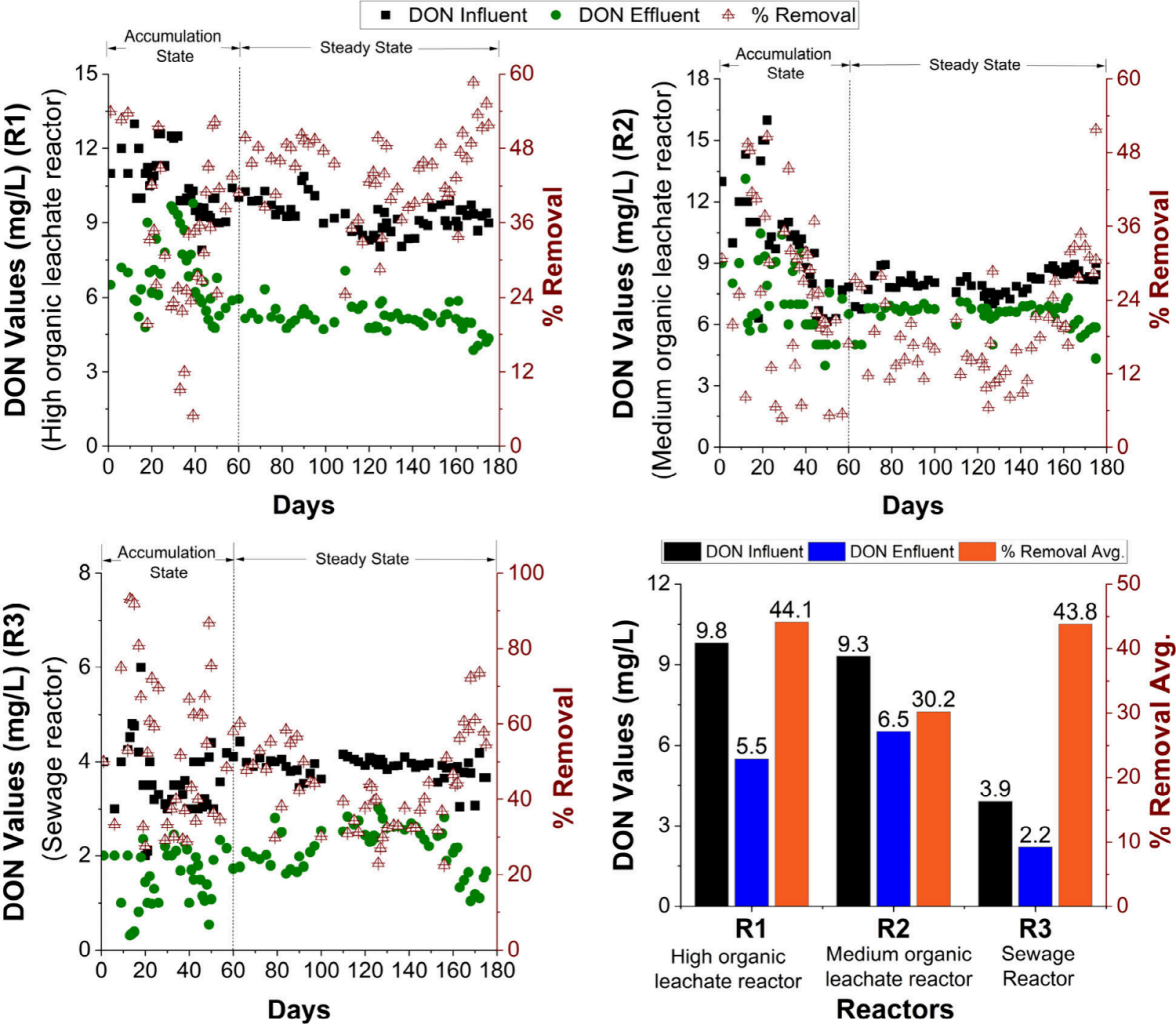
Influent and effluent DON values with respective removal efficiency for R1 (high organic leachate reactor), R2 (medium organic leachate reactor), and R3 (sewage reactor), respectively.

The extent of DON removal is closely linked to the balance between bioavailable and refractory organic nitrogen fractions,^40^ which can be indirectly assessed using the influent C/N ratio. The influent C/N ratios for R1 (high-organic-leachate reactor), R2 (medium-organic-leachate reactor), and R3 (sewage reactor) were 23.2, 7.9, and 8.8, respectively. At the lower C/N ratio, R2 (medium organic leachate reactor) exhibited higher effluent DON concentrations and reduced removal efficiency, whereas R1 (high organic leachate reactor) achieved more effective DON removal under a higher C/N ratio. This pattern supports the interpretation that carbon-rich conditions enhance microbial access to labile DON fractions, facilitating greater transformation during the biological treatment. These findings are consistent with a previous pilot-scale biofilter study treating secondary effluent, which reported improved DON removal under higher influent C/N ratios.^9^

Collectively, these results indicate that influent organic composition and carbon availability play critical roles in determining DON persistence during BNR, providing a mechanistic basis for the subsequent evaluation of DON molecular composition and microbial transformation pathways.

### 3.3 Effluent Organic Compounds Characterization

#### 3.3.1 Molecular Composition of Effluent Organics by FTICR-MS

FTICR-MS identified 10,550, 9,897, and 8,331 molecular formulas for organic compounds in R1 (high-organic leachate reactor), R2 (medium-organic leachate reactor), and R3 (sewage reactor), respectively. Van Krevelen plots (Figure 5), based on H/C (hydrogen-to-carbon) and O/C (oxygen-to-carbon) molar ratios and considering compounds with relative abundance > 0.25%,^31^ were used to classify molecular formulas into functional groups.^43^ This approach enables comparison of organic matter composition and potential bioavailability among reactor effluents. Van Krevelen analysis revealed a highly complex mixture of organic compounds in all effluents. Leachate–sewage blend effluents from R1 (high organic leachate reactor) and R2 (medium organic leachate reactor) exhibited broadly similar molecular distributions, whereas the sewage-only effluent from R3 showed a distinct compositional pattern, reflecting differences in influent sources and transformation pathways during biological treatment.

**Figure 5:**
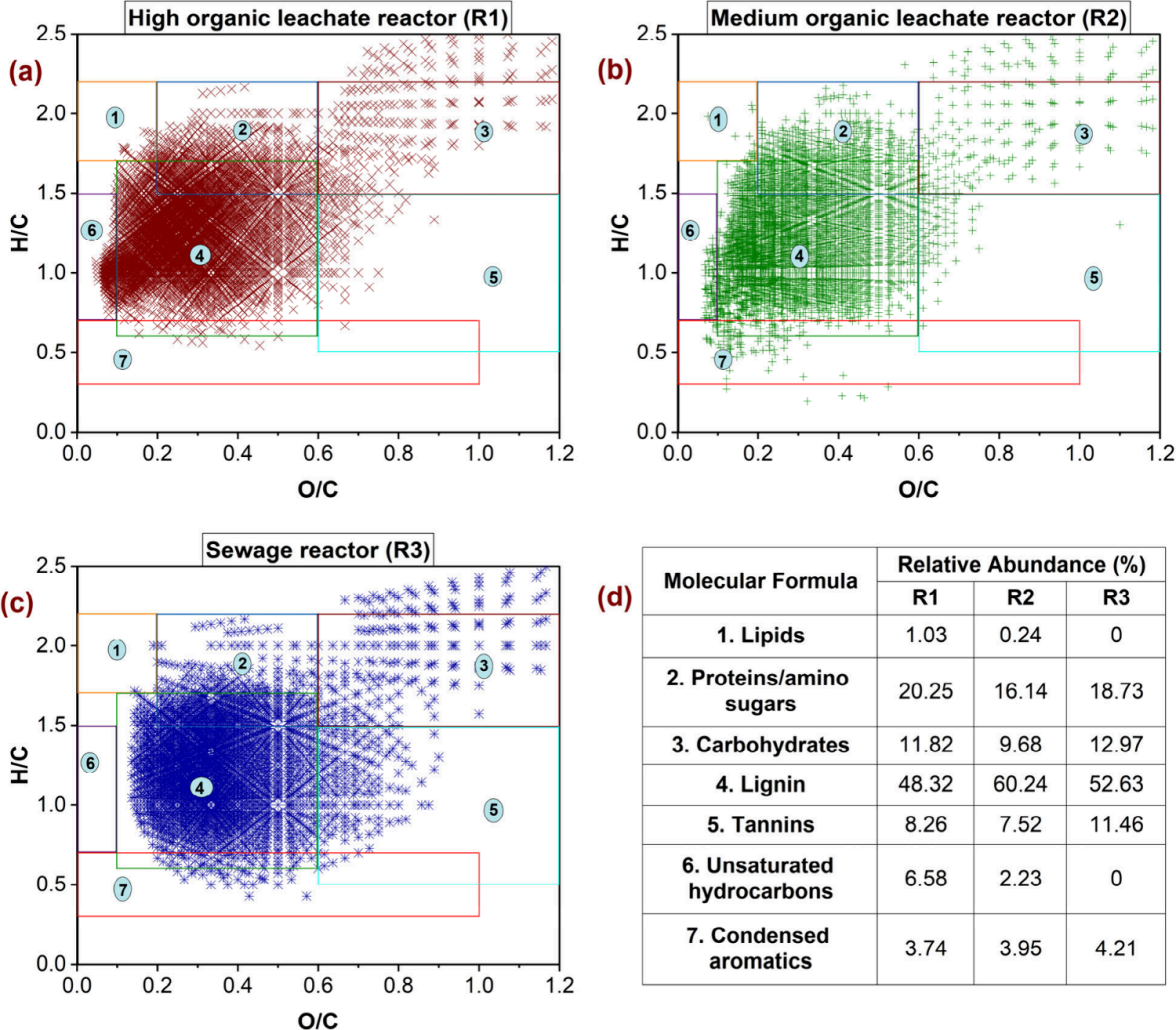
Van Krevelen diagrams of effluent organics for R1 (high organic leachate reactor) (a), R2 (medium organic leachate reactor) (b), and R3 (sewage reactor) (c), and the relative abundances of organic compounds (d). FTICR-MS elemental ratios represent different chemical compound groups.^9^ Each Van Krevelen plot is divided into seven zones, with labeled circles indicating (1) lipids (O/C = 0–0.2, H/C = 1.7–2.22), (2) proteins/amino sugars (O/C = 0.2–0.6, H/C = 1.5–2.23), (3) carbohydrates (O/C = 0.6–1.2, H/C = 1.5–2.24), (4) lignin (O/C = 0.1–0.6, H/C = 0.6–1.75), (5) tannins (O/C = 0.6–1.2, H/C = 0.5–1.56), (6) unsaturated hydrocarbons (O/C = 0–0.1, H/C = 0.7–1.57), and (7) condensed aromatics (O/C = 0–1, H/C = 0.3–0.7). R1 received 0.8% L-A (high organic leachate) with sewage as influent, R2 received 3% L-B (medium organic leachate) with sewage as influent, and R3 was fed exclusively with sewage.

In the R1 (high-organic-leachate-reactor) effluent, lignin-like compounds constituted the most abundant group, followed by proteins/amino sugars, carbohydrates, tannins, unsaturated hydrocarbons, condensed aromatics, and lipids. R2 (medium organic leachate reactor) effluent exhibited a similar distribution, with lignin > proteins/amino sugars > carbohydrates > tannins > condensed aromatics > unsaturated hydrocarbons > lipids. Although R1 (high-organic-leachate-reactor) and R2 (medium-organic-leachate-reactor) effluents shared similar compound classes, their relative abundances differed, indicating differences in DON quality rather than quantity. Specifically, R1 (high organic leachate reactor) contained a higher proportion of unsaturated hydrocarbons than condensed aromatics, whereas R2 (medium organic leachate reactor) showed the opposite trend, consistent with a more aromatic and refractory organic pool in the medium organic leachate. In contrast, no lipids or unsaturated hydrocarbons were detected in the sewage effluent from R3 (sewage reactor), consistent with previous reports on BNR-treated sewage effluent.^8^ Carbohydrates, tannins, and condensed aromatics were relatively more abundant in R3 (sewage reactor) effluent than in R1 (high organic leachate reactor) and R2 (medium organic leachate reactor), reflecting sewage-derived organic matter characteristics.

Lignin-like compounds and proteins/amino sugars dominated the effluent organic matter in all reactors (Figure 5d), with lignin accounting for 48.32% (R1, high organic leachate reactor), 60.24% (R2, medium organic leachate reactor), and 52.63% (R3, sewage reactor)), and proteins/amino sugars contributing 20.25% (R1, high organic leachate reactor), 16.14% (R2, medium organic leachate reactor), and 18.73% (R3, sewage reactor). This compositional distribution is consistent with previous studies of BNR effluents, where protein/amino sugar–like and lignin-like compounds are commonly reported as dominant DON fractions.^8,9,41,10^ R2 (medium organic leachate reactor) exhibited the highest lignin content, while R1 (high organic leachate reactor) and R3 (sewage reactor) showed greater relative contributions from proteins/amino sugars, with R1 (high organic leachate reactor) being the highest. Proteins/amino sugar–like compounds are generally associated with labile, bioavailable DON, whereas lignin-like compounds are considered more refractory due to their aromatic structure and resistance to microbial degradation.^44,39^ Accordingly, the higher protein/amino sugar fraction in R1 (high organic leachate reactor) effluent suggests greater DON bioavailability, followed by R3 (sewage reactor) effluent, whereas the elevated lignin content in R2 (medium organic leachate reactor) effluent indicates a more recalcitrant DON pool. This molecular-level distinction provides a mechanistic explanation for the higher DON removal observed in R1 (high organic leachate reactor) and R3 (sewage reactor) compared to R2 (medium organic leachate reactor) in Sections 3.1 and 3.2 and establishes a compositional basis for subsequent evaluation of microbial transformation and ecological response.

#### 3.3.2 ^13^C NMR and FTIR Spectroscopy of DON/DOM Compounds

^13^C NMR analysis indicated that biological treatment substantially removed or transformed most influent organic components, while a persistent signal was observed in the chemical shift region δ of 164–166 ppm (ppm), attributed to carbonyl carbons, remained detectable, suggesting the presence of recalcitrant organic structures. ^13^C NMR details can be found in S.I. section 6. FTIR spectra further revealed compositional differences between influent and effluent organic matter. Influent samples exhibited characteristic features associated with ammonium, nitrate/nitrite, and amine functional groups, whereas effluent samples showed attenuated or altered absorbance patterns, indicating compound removal and transformation during SBR treatment. FTIR results therefore corroborate FTICR-MS and ^13^C NMR findings, demonstrating selective removal of labile organic components and enrichment of refractory functional groups. FTIR details can be found in S.I. section 7.

Collectively, molecular and spectroscopic analyses indicate that biological treatment preferentially removes bioavailable DON, leaving effluents relatively enriched in refractory organic nitrogen, particularly in medium-organic leachate systems. These compositional differences are expected to influence microbial utilization and short-term ecological responses, as examined in subsequent sections.

### 3.4 Microbial Community Distribution Analysis of the Activated Sludge

Microbial community analysis (Figure 6) showed distinct differences among reactors, reflecting variations in influent nitrogen and organic carbon composition. R2 (medium organic leachate reactor) exhibited the highest relative abundance of ammonia-oxidizing bacteria (AOB, 11.32%) and nitrite-oxidizing bacteria (NOB, 8.39%), followed by R1 (high organic leachate reactor) (AOB 4.76%, NOB 3.7%) and R3 (sewage reactor) (AOB 3.3%, NOB 3.2%). Elevated influent NH_4_^+^-N in R2 (medium organic leachate reactor) likely promoted AOB growth and subsequent NO_2_^–^-N production, supporting NOB enrichment.^45–47^ In all reactors, AOB abundance exceeded that of NOB, consistent with sequential nitrification kinetics.^45^

**Figure 6:**
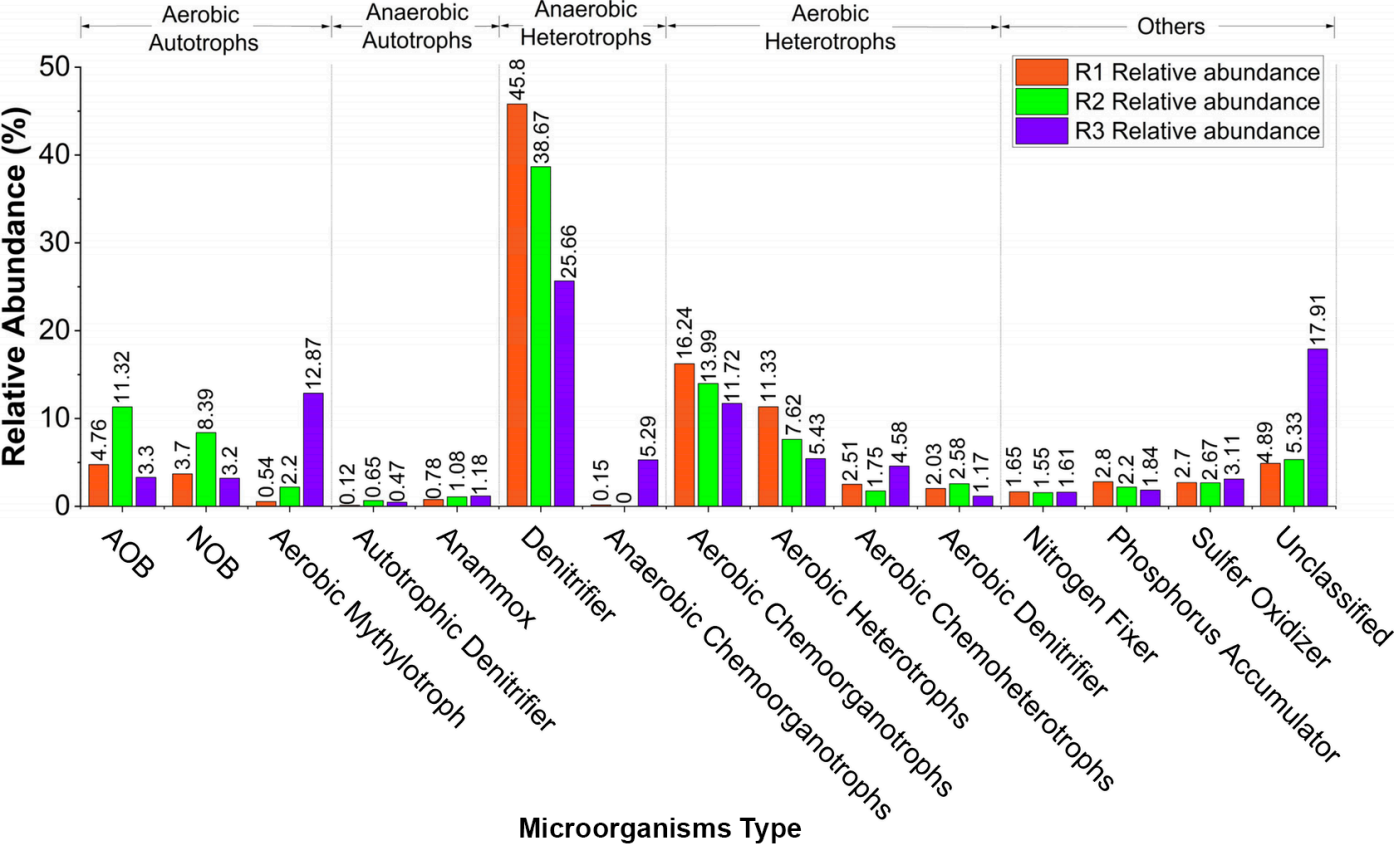
Microbial community analysis of the activated sludge samples from R1 (high organic leachate reactor), R2 (medium organic leachate reactor), and R3 (sewage reactor) reactors, including microorganism types and relative abundances.

In contrast, R3 (sewage reactor) was characterized by a high abundance of aerobic methylotrophs (12.87%), reflecting selection driven by methanol addition.^48^ *Methylophilus* (5.34%) and *Methylotenera* (4.24%) dominated the methylotrophic community (Figure S14), consistent with previous studies reporting enrichment of *Methylophilaceae* in laboratory-scale SBRs and identifying *Methylophilus* as a dominant methanol-utilizing denitrifier, highlighting the role of methanol-fed systems in selecting specialized methylotrophic populations.^49^ *Hyphomicrobium* was present at low abundance (<1.5%) (Figure S14), consistent with a report indicating its minor role in methanol-driven denitrification relative to *Methylophilus*-dominated communities.^50^

Anaerobic or aerobic heterotrophic populations, including denitrifiers and chemoorganotrophs, were most abundant in R1 (high organic leachate reactor) (denitrifiers 45.8%, chemoorganotrophs 16.24%), followed by R2 (medium organic leachate reactor) and R3 (sewage reactor). As heterotrophs rely on organic carbon for growth,^51,52^ their enrichment in R1 (high-organic leachate reactor) reflects the higher availability of bioavailable organic matter in high-organic leachate. This microbial structure is consistent with the higher COD removal observed in R1 (high-organic-leachate reactor) and supports enhanced nitrogen transformation through coupled heterotrophic denitrification.^53,54^ In contrast, R3 (sewage reactor) contained a higher fraction of unclassified microorganisms, suggesting a lower functional specialization.

At the phylum level (Figure S13), *Proteobacteria, Bacteroidetes, Verrucomicrobia, Acidobacteria, Parcubacteria, Firmicutes*, and *Chloroflexi* collectively accounted for over 80% of the microbial community in R1 (high organic leachate reactor) and approximately 70% in R2 (medium organic leachate reactor) and R3 (sewage reactor). This composition is consistent with previous SBR studies treating municipal wastewater: Hu et al. (2020) reported *Proteobacteria, Bacteroidetes, Verrucomicrobia, Acidobacteria*, and *Chloroflexi* as dominant phyla in sewage-fed SBRs, while a similar dominance was also observed by Yuan et al. (2016).^8,55^ *Proteobacteria* contribute significantly to nitrogen metabolism, whereas *Bacteroidetes* are closely associated with organic matter degradation, particularly protein-rich dissolved organic matter, highlighting their combined importance in DON transformation within wastewater treatment systems.^56–59^ *Proteobacteria* and *Bacteroidetes* dominated R1 (high organic leachate reactor), consistent with its higher organic carbon availability and greater DON bioavailability,^60^ whereas *Verrucomicrobia* and *Parcubacteria* were more abundant in R2 (medium organic leachate reactor), reflecting a more refractory organic pool.^61^ *Acidobacteria* showed higher representation in R3 (sewage reactor), aligning with its lower organic loading.

At the genus level (Figure S14), *Zoogloea, Thermomonas, Lewiella*, and *Terrimonas* were most abundant in R1 (high organic leachate reactor), contributing to COD reduction and NO_3_^–^-N removal.^54,55^ *Nitrosomonas*, *Nitrosococcus*, and *Nitrosospira* are present in both R1 (high organic leachate reactor) and R2 (medium organic leachate reactor), with R2 (medium organic leachate reactor) also having *Nitrobacter*, indicating higher AOB abundance in R2 (medium organic leachate reactor) than R1 (high organic leachate reactor), consistent with the higher influent NH_4_^+^-N load. In contrast, R3 (sewage reactor) contained only *Nitrosococcus*, reflecting its lower influent NH_4_^+^-N.

Few studies explored the connection between microbes, DON, bioavailable DON, and DIN.^62^ *Proteobacteria*, *Chloroflexi*, *Planctomycetes*, and *Bacteroidetes* significantly affect bioreactor effluent DON variation.^63^ *Bacteroidetes*, previously demonstrated to degrade high-molecular-mass DOM, particularly proteins,^8^ are more abundant in R1 (high organic leachate reactor) (27.39%) than in R2 (medium organic leachate reactor) (20.63%) and R3 (sewage reactor) (16.92%). Their enrichment aligns with the higher protein/amino sugar fraction and greater DON removal observed in R1 (high organic leachate reactor), indicating active microbial turnover of bioavailable DON.^63^ The enriched *Firmicutes* in this study are known to enhance enzymatic versatility,^64^ suggesting that their presence may contribute to broader metabolic pathways driving nitrogen and organic carbon removal in BNR systems. Additionally, the coexistence of *Actinobacteria* with *Proteobacteria* and *Chloroflexi* in the study has also been reported to synergistically facilitate pollutant removal,^64^ which aligns with the simultaneous removal of TN and DON observed in this study. Finally, *Planctomycetes*, which are positively correlated with carbohydrate-like DON compounds,^63^ were enriched in R3 (sewage reactor), consistent with the higher carbohydrate fraction in this reactor effluent.

Overall, influent organic composition shapes the microbial community structure, which in turn controls DON transformation and bioavailability, linking effluent DON composition to subsequent ecological responses.

### 3.5 *In Situ* Algal Bioassay

Biological treatment effectively removed dissolved inorganic nitrogen (DIN; NH_4_^+^-N and NO_3_^–^-N), resulting in DON-dominated effluents from R1 (high-organic leachate reactor), R2 (medium-organic leachate reactor), and R3 (sewage reactor), whereas raw leachates (L-A (high organic leachate) and L-B (medium organic leachate)) remained rich in NH_4_^+^-N. Phytoplankton responses were assessed using HPLC-based photopigment analysis, including chlorophyll a (total biomass), chlorophyll b (green algae), fucoxanthin (diatoms), alloxanthin (cryptophytes), peridinin (dinoflagellates), myxoxanthophyll (cyanobacteria), and zeaxanthin (cyanobacteria and other taxa).^65^ Figure 7 summarizes photopigment responses during the bioassay.

**Figure 7:**
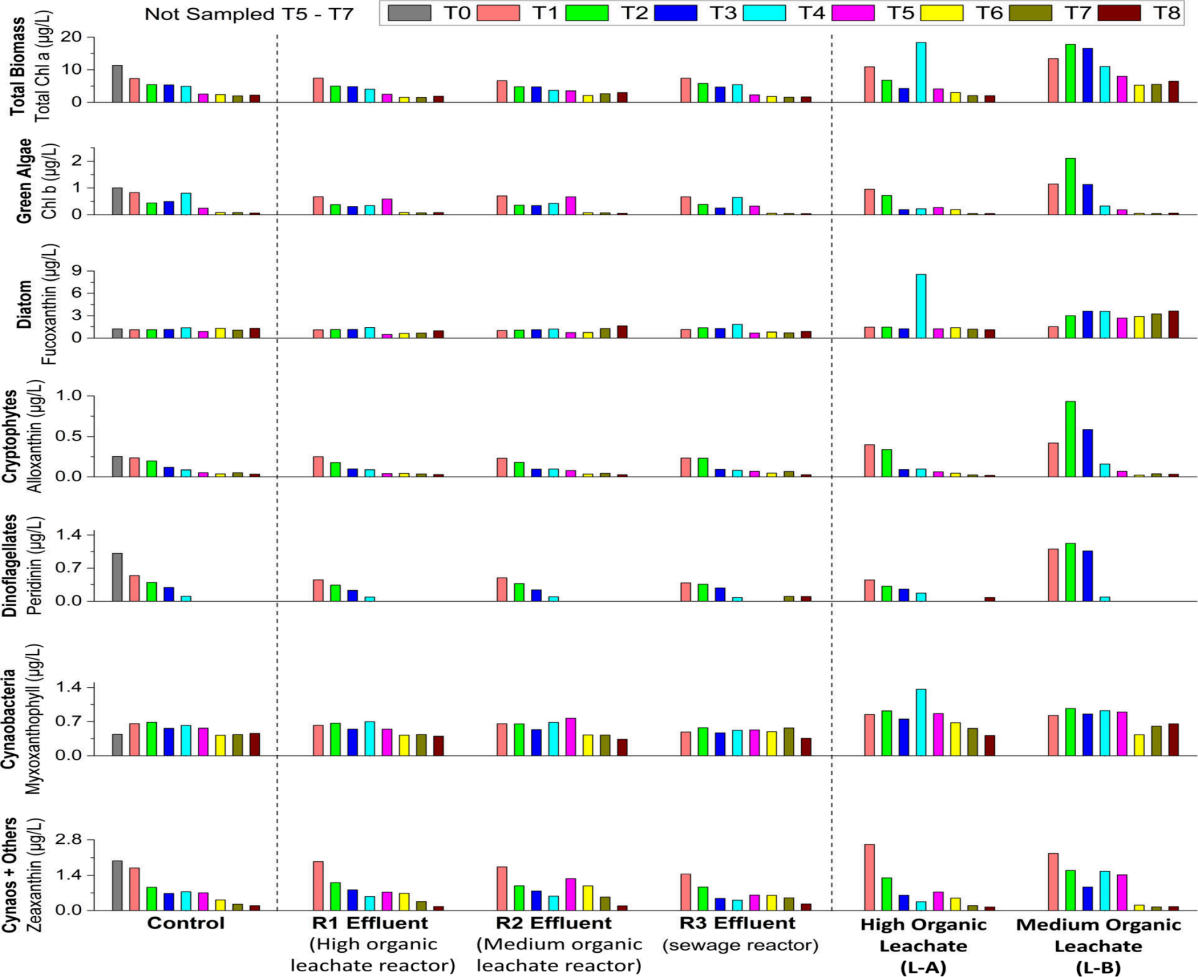
Detected algae species for SBRs effluents R1 (high organic leachate reactor), R2 (medium organic leachate reactor), and R3 (sewage reactor) and raw leachates L-A (high organic leachate) and L-B (medium organic leachate) in HPLC analysis of phytoplankton group photopigment responses during the September 2022 bioassay. The bars represent the mean of the triplet tests for each sample. (T0 - day 1, T1 - day 2, T2 - day 3, T3 - day 4, T4 - day 5, T5- day 8, T6 - day 9, T7 - day 10, T8 - day 11).

Chlorophyll a (Chl-a), representing total phytoplankton biomass, showed similar growth patterns among treated effluents and the control, with no significant differences across R1 (high organic leachate reactor), R2 (medium organic leachate reactor), and R3 (sewage reactor) effluents. Although molecular analyses indicated differences in DON composition among reactors, chlorophyll a (Chl-a) showed similar growth patterns across all treatments, suggesting that a large fraction of effluent DON was not readily bioavailable within the short term (11-day) incubation period. Peak Chl-a concentrations in treated effluents remained below ∼8 μg/L (e.g., 7.5 μg/L in R1 (high organic leachate reactor) at T1), whereas raw leachates exhibited substantially higher responses. In particular, L-B (medium organic leachate) reached Chl-a peaks of 17.8 and 16.6 μg/L at T2 and T3, respectively, while L-A (high organic leachate) peaked at 18.4 μg/L at T4. These results indicate that DON-dominated effluents provided limited short-term algal stimulation compared to DIN-rich raw leachates. This limited stimulation is attributable to the refractory nature of DON in treated leachate and sewage effluents, where nitrogen is predominantly present as DON.^15^ Consistent with this observation, temporal changes in DON during the 11-day bioassay showed low apparent algal utilization rates of approximately 11.0, 2.5, and 6.9 μg/L/day for R1 (high organic leachate reactor), R2 (medium organic leachate reactor), and R3 (sewage reactor), respectively, further confirming the limited short-term bioavailability of effluent DON (see SI section 10 for more details). In contrast, raw leachates contain both bioavailable and refractory DON as well as DIN, among which DIN and labile DON support enhanced algal growth.^15,66^

Chlorophyll b (green algae) responses in treated effluents followed trends similar to the control but remained consistently lower across all time points (T0–T8), with early fluctuations (T0–T5) followed by an overall decline (Figure 7). In contrast, raw leachate L-B (medium organic leachate) showed higher stimulation, reaching a peak chlorophyll b concentration of 2.1 μg/L at T2, exceeding both L-A (high organic leachate) and treated effluents. Fucoxanthin (diatoms) concentrations in treated effluents did not differ significantly from the control, whereas L-B (medium organic leachate) generally exceeded L-A (high organic leachate), except at T4 when L-A exhibited a transient peak of 8.54 μg/L. Alloxanthin (cryptophytes) concentrations in treated effluents were comparable to the control, while L-B (medium organic leachate) peaked at 0.93 μg/L at T2. Peridinin (dinoflagellates) responses were limited after T4; treated effluents showed reduced concentrations relative to the control but similar temporal patterns, whereas L-B (medium organic leachate) consistently exhibited the highest levels. Myxoxanthophyll (cyanobacteria) displayed uniform patterns across treatments, with lower concentrations in treated effluents than in untreated leachates. Zeaxanthin responses largely mirrored control patterns, with untreated L-B (medium organic leachate) showing enhanced early stage growth.

Nutrient-spiked controls confirmed nitrogen limitation in the Neuse River Estuary bioassay water (Figure S15). Both NO_3_^–^ and NO_3_^–^+P additions produced similar algal biomass responses, whereas the P-only treatment showed minimal growth, indicating nitrogen limitation. Based on the nutrient data (Figure S16), TDN dynamics varied across treatments over time. TDN increased from T0 to T3 in R1 (high organic leachate reactor) and R2 (medium organic leachate reactor) and then fluctuated, whereas R3 (sewage reactor) initially declined. By the final sampling point, TDN in R1 (high organic leachate reactor) and R3 (sewage reactor) was lower than initial levels, whereas R2 (medium organic leachate reactor) remained elevated. Raw leachate L-A (high organic leachate) showed stable TDN, while L-B (medium organic leachate) decreased over time. In spiked samples, TDN was dominated by DIN and progressively consumed by algae; NH_4_^+^ remained low except in L-B, and NO_3_^–^ was fully depleted in NO_3_^–^-spiked treatments. Silica and phosphate showed no consistent depletion, indicating they were not limiting.

Overall, these results demonstrate limited short-term algal bioutilization of biologically treated effluents dominated by refractory DON, whereas DIN-rich raw leachates elicited pronounced phytoplankton responses. This indicates that SBR treatment effectively reduces the algal stimulatory potential of landfill leachate–derived nitrogen, lowering short-term eutrophication risk in receiving waters.

## 4 Conclusion

This study examined BNR cotreatment of landfill leachate (high and medium organic content) with municipal sewage, evaluating treatment performance, DON characteristics, microbial dynamics, and ecological implications.

Across all SBRs, NH_4_^+^-N removal approached 98–99% and TN removal 87–92%, with low residual NO_3_^–^-N achieved via methanol-aided denitrification. However, DON persisted as the dominant nitrogen fraction (∼70–80% of effluent TN). The high-organic leachate blend (R1) removed DON more effectively than the medium-organic blend (R2), consistent with FTICR-MS evidence that R1 (high organic leachate reactor) effluent contained more protein/amino-sugar–like (more bioavailable) DON whereas R2 (medium organic leachate reactor) was enriched in lignin-like, refractory compounds.

^13^C NMR and FTIR analyses further confirm that biological treatment preferentially removes labile compounds while leaving persistent carbonyl-associated structures (164–166 ppm), highlighting challenges in DON degradation. Parallel shifts in microbial communities (heterotrophic denitrifiers in R1 (high organic leachate reactor) (45.8%); stronger AOB/NOB enrichment in R2 (medium organic leachate reactor) (11.32%/8.39%); methylotrophs in R3 (sewage reactor) (12.87%)) aligned with influent chemistry and process performance. The dominance (>80% for R1 (high organic leachate reactor) and R2 (medium organic leachate reactor) and >70% for R3 (sewage reactor)) of seven phyla (*Proteobacteria*, *Bacteroidetes*, *Verrucomicrobia*, *Acidobacteria*, *Parcubacteria*, *Firmicutes*, *Chloroflexi*) in SBR systems indicates their crucial roles in organic degradation and nitrogen cycles.

In situ estuarine bioassays indicated that treated effluents—despite being DON-rich—produced limited short-term phytoplankton stimulation, while raw leachates rich in DIN and bioavailable DON stimulated algal pigments, underscoring N-limitation and the disproportionate role of DIN in acute bloom risk. This finding highlights the limited short-term bioavailability of effluent DON.

Collectively, these results demonstrate that (i) BNR cotreatment is effective at DIN control, thereby mitigating near-field eutrophication potential, but (ii) effluent DON remains a persistent and composition-dependent nitrogen pool whose composition is source-dependent and may influence downstream ecosystems over longer residence times. We recommend that utilities cotreating leachate (1) monitor DON quantity and composition alongside conventional DIN metrics and (2) consider polishing steps targeting refractory DON where receiving waters are sensitive (e.g., advanced oxidation or adsorptive treatments). Future work should focus on longer-term in situ bioassays and evaluation of treatment strategies to enhance DON removal and better align wastewater management with watershed-scale nutrient control.

## Supplementary Material



## Notes

Biological reactor setup and operation, FTICR-MS method, *in situ* algal bioassay method, Microbial community analysis method, additional SBR operation results, ^13^C NMR spectroscopic analysis, FTIR spectroscopic analysis, additional microbial community analysis results, additional in situ algal bioassay results, DON utilization rate by algae, statistical analysis of denitrification improvement (PDF)
