# Supplementary material for: Fate and Transformation of Landfill Leachate Dissolved Organic Nitrogen and Its Implications for Estuarine Algal Growth
Source: ACS ES T Water. 2026 May 18;6(6):3769–81. doi: 10.1021/acsestwater.6c00163 (PMC13270524; doi:10.1021/acsestwater.6c00163)
Supplement: Supplementary file 1 [file ew6c00163_si_001.pdf]

Fate and Transformation of Landfill Leachate Dissolved Organic Nitrogen and Its Implications for Estuarine Algal Growth

Md Ashik Ahmed[1], Brian Brazil[2], Wenzheng Yu[3], Lifeng Zhang[1], Hans W. Paerl[4,], Renzun Zhao[5,*]*

[1]Nanoengineering Department, Joint School of Nanoscience and Nanoengineering, North Carolina A&T State University, 2907 E Gate City Blvd, Greensboro, NC 27401, United States

[2]Waste Management Inc. Gaithersburg, MD 20878, United States

[3]State Key Laboratory of Environmental Aquatic Chemistry, Research Center for Eco-Environmental Sciences, Chinese Academy of Sciences, Beijing 100085, China

[4]Department of Earth, Marne and Environmental Sciences, Institute of Marine Sciences, University of North Carolina Chapel Hill, Morehead City, NC, 28557, United States

[5]Civil, Architectural and Environmental Engineering Department, North Carolina A&T State University, Greensboro, NC 27411, United States

*Corrensponding authors: Renzun Zhao (rzhao@ncat.edu); Hans W. Paerl (hans_paerl@unc.edu)

SUPPORTING INFORMATION

S1. Biological reactor setup and operation

The experiment uses three SBRs (R1, R2, and R3) made from acrylic plastic sheets with a total volume of 5 liters and a working volume of 3 liters, having the dimensions 7.10 inches in length x width x height. Each reactor was fitted with a mechanical stirrer, air diffuser, water heater, pH, dissolved oxygen (DO), and ion selective electrode (ISE) probes as needed. SBR operation started on March 10, 2022, and during the startup period, all the reactors were only fed with sewage. After good nitrification was achieved, landfill leachates L-A and L-B were added to R1 and R2,

respectively. Reactor R3 continued with only sewage feeding. From day 12 onwards, the dosing of leachate reached 0.8% for L-A and 3% for L-B. The Reactors were operated with these leachate percentages until the end of the study period (August 30, 2022). The study was run for a total of 175 days, with the first 60 days being in an accumulation state and the second 115 days being in a steady state condition. Methanol, as an organic carbon source, was introduced for all the reactors from Day 12 for complete denitrification to the rest of the study period. The airflow was maintained at a rate of 0.8, 0.5, and 0.4 L/min for reactors R1, R2, and R3, respectively, to keep the DO level in the aerobic phase between 1 and 2 mg/L throughout the study period. Since the operation began, effluent samples were taken and analyzed daily. Each sample was filtered using a 0.45 µm GF/F glass microfiber filter before testing.

S2. FTICR-MS method and sample preparation

To examine DON molecular composition, advanced analytical methods, such as ultrahigh resolution FTICR-MS can be used.[9] The integration of negative-ion electrospray ionization (ESI) inlet systems with mass spectrometers of varying resolutions can significantly enhance the capacity to analyze and identify dissolved organic matter (DOM) at the molecular level in aqueous environmental samples.[10] FTICR-MS accurately measures the mass of thousands of organic molecules in landfill leachate samples, assigning molecular formulas that reveal their transformation during wastewater treatment.[11] Unlike most mass analyzers, which cannot resolve thousands of molecules differing by less than an electron's mass, 21T FTICR-MS exceeds 1,500,000 resolving power, allowing precise molecular separation and accurate elemental composition assignments across a broad mass range (150<m/z<1200) with sub-ppm accuracy.[12] Following solid-phase extraction, negative-ion ESI enhances organic carbon detection by selectively deprotonating acidic species for analysis with 21T FTICR-MS, the most sensitive and high-resolution mass analyzer to date.[13]

S2.1. Sample preparation

High-performance liquid chromatography (HPLC)-grade (Sigma-Aldrich Chemical Co., St. Louis, MO) solvents and solid-phase extraction (SPE) extracts were used for FTICR-MS without negative ion electrospray ionization (ESI).

Sample Collection

For sample collection, acid-washed Nalgene high-density polyethylene (HDPE) or polycarbonate (PC) bottles were used. Each container was rinsed three times with the sample before final collection to avoid contamination. Samples were then collected and immediately prepared for filtration to maintain sample integrity.

Sample Filtration

The filtration process involved baked glass microfiber (GF/F) filters, Supor membranes, and acid-washed filter holders, hoses, and a peristaltic pump. Samples were homogenized by gentle shaking before filtration. The filtration system was flushed with Milli-Q water, and hoses were connected to both a waste container and a bottle of Milli-Q water. After flushing, the sample was filtered, and collection bottles were rinsed with the initial filtrate before collecting the final sample. Filtered samples were acidified to pH 2 and refrigerated until ready for the cartridging step. All materials used in filtration were thoroughly cleaned and air-dried at room temperature.

Cartridging

For the cartridging step, polymeric porous layer (PPL) cartridges, polytetrafluoroethylene (PTFE) tubing, a vacuum manifold, and acid-washed hoses were used. Samples were brought to room temperature, and the hoses and cartridges were connected to the vacuum manifold. Cartridges were cleaned by running 15 mL each of methanol and acidified Milli-Q water (pH 2) through them. The cartridges were then attached to the sample bottles, and 100 mL of each sample was run through. Acidified water was used afterward to remove any remaining salts. Cartridges were dried in a fume hood using ultrapure nitrogen gas.

Eluting, Concentrating, and Storing

Elution involved labeled glass tubes and an evaporator. Samples were eluted with methanol, taking care to avoid spills or contamination. The volume was then reduced under nitrogen gas in an evaporator to approximately 3-4 mL. The concentrated extracts were transferred to final vials, volumes adjusted with ultrapure nitrogen, tightly capped, and stored in a freezer until analysis.

S2.2. Instrumentation: ESI Source

The sample solution was infused via a microelectrospray source [1] (50 μm i.d. fused silica emitter) at 500 nL/min by a syringe pump. Typical conditions for negative ion formation were: emitter voltage, -2.4-2.9 kV; S-lens RF level: 45%; and heated metal capillary temperature, 350 ° C.

S2.3. Instrumentation: 21 T FT-ICR MS.

DOM extracts were analyzed with a custom-built hybrid linear ion trap FT-ICR mass spectrometer equipped with a 21 T superconducting solenoid magnet [2,3]. Ions were initially accumulated in an external multipole ion guide (1-5 ms) and released m/z-dependently by decrease of an auxiliary radio frequency potential between the multipole rods and the end-cap electrode [4]. Ions were excited to m/z-dependent radius to maximize the dynamic range and number of observed mass spectral peaks (32-64%) [4], and excitation and detection were performed on the same pair of electrodes [5]. The dynamically harmonized ICR cell in the 21 T FT-ICR is operated with a 6 V trapping potential [4,6]. Time-domain transients of 3.1 seconds were acquired with the Predator data station that handled excitation and detection only, initiated by a TTL trigger from the commercial Thermo data station, with 100 time-domain acquisitions averaged for all experiments [7]. Mass spectra were phase-corrected [8] and internally calibrated with 10-15 highly abundant homologous series that span the entire molecular weight distribution based on the "walking" calibration method [9]. Experimentally measured masses were converted from the International Union of Pure and Applied Chemistry (IUPAC) mass scale to the Kendrick mass scale [10] for rapid identification of homologous series for each heteroatom class (i.e., species with the same CcHhNnOoSs content, differing only be the degree of alkylation) [11]. For each elemental composition, CcHhNnOoSs, the heteroatom class, type (double bond equivalents, DBE = number of rings plus double bonds to carbon, DBE = C –h/2 + n/2 +1)[12] and carbon number, c, were tabulated for subsequent generation of heteroatom class relative abundance distributions and graphical relative-abundance weighted images and van Krevelen diagrams. Peaks with signal magnitude greater than 6 times the baseline root-mean-square (RMS) noise at m/z 500 were exported to peak lists, and molecular formula assignments and data visualization were performed with PetroOrg © software [12]. Molecular formula assignments with an error >0.5 parts-per-million were discarded, and only chemical classes with a combined relative abundance of ≥0.15% of the total were considered.

S3. *In-situ* algal bioassay method

For the algal bioassays, 4L volume 0.01N HCL followed by deionized water-rinsed polyethylene (PE) Cubitainers were used as incubation vessels. Cubitainers are chemically inert and 85% photosynthetically active radiation (PAR) transparent, were deployed in floating corrals in a pond to replicate natural light and temperature conditions outside the University of North Carolina at Chapel Hill - Institute of Marine Science (UNC-IMS), Morehead City, NC, USA facility (Figure

S1).[13,14] Corrals were covered with a layer of neutral density screening, which reduced the incident light by 20%, avoiding photoinhibition. Cubitainers contained a blend of effluent samples and estuarine surface water collected from site 100 in the Neuse River Estuary, NC, USA, through the UNC-IMS ModMon Project (https://paerllab.web.unc.edu/modmon/). Estuarine water was pre-filtered through a 200µm Nitex mesh to exclude filter-feeding zooplankton. Site 100 is strategically located just upstream of the estuarine bend, in oligo to mesohaline N-limited waters.[15] Each treatment involved using Neuse River Estuary water, effluent samples, and nutrient spikes in triplicate in Cubitainers, which were continuously mixed in a holding tank and thoroughly stirred before subsampling to ensure homogeneity. Bioassay was performed in September 2022 and lasted 11 days with nine (T0 to T8) sampling time points.

The bioassay tested the bioreactivity (as net algal growth determined by diagnostic photopigments) of R1 and R2 effluent leachates, L-A and L-B raw leachates, R3 effluent sewage, and nitrate (NO_3), phosphorous (P), and NO_3+P nutrient-spiked samples. There are control samples as well with the Neuse River water. Comparing R1, R2, and R3 showed that leachate, sewage, and organic content influenced algal growth. Effluent samples added to Cubitainers maintained consistent TN levels (approximately 30 µM N/L) in all treatments. At the initiation of the bioassay, dissolved inorganic carbon at the site was 19.96 mg C/L, with an additional 10 mg C/L of $NaHCO_3$ added uniformly to all Cubitainers to prevent carbon limitation across controls and treatments. Subsamples from Cubitainers were collected semiweekly for chlorophyll a and HPLC diagnostic pigment analysis of major algal groups[16] and microscopic phytoplankton community composition in response to leachate treatments. The experiment used known dissolved inorganic nitrogen (DIN) additions and included untreated controls for comparative analysis. Figure S1 shows the bioassay setup, including Cubitainers and pond incubation to mimic estuary conditions.

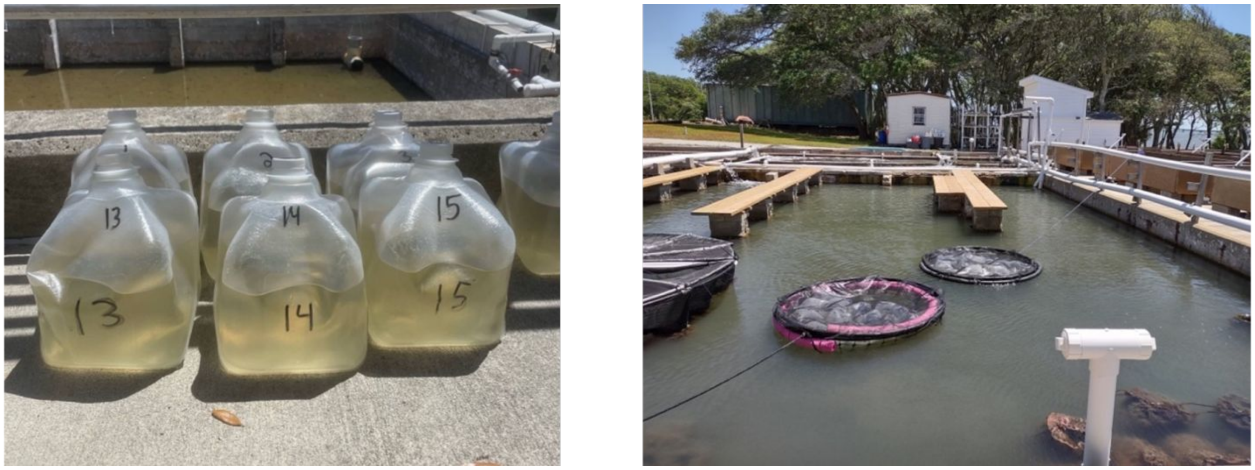

Figure S1. Algal bioassay setup; prepared Cubitainers on the left and the pond incubation on the right.

S4. Microbial community analysis method

At the end of the evening cycle, activated sludge samples were taken from reactors R1, R2, and R3 on day 150 of the SBRs' operation. Samples were transferred into sterile 100 mL high-density polyethylene (HDPE) bottles, ensuring no external contamination. This allowed for sludge analysis following a full operational cycle, revealing the microbial and chemical characteristics that had developed in each reactor.

16S Metagenomic Sequencing and qPCR

Samples were shipped to Microbial Insights, Inc. (Knoxville, TN) for molecular analysis. DNA extractions were performed on the same day as the receipt, following the manufacturer's instructions. 16S metagenomic sequencing was conducted using bacterial primers designed for the high throughput Illumina MiSeq platform. Taxonomic classification of 16S-rRNA amplicon reads was performed with the Illumina 16S Metagenomics application, which utilizes a curated version of the Green Genes taxonomic database and a high-performance implementation of the Ribosomal Database Project (RDP) Classifier algorithm [17]. Phylogenetic operational taxonomic unit (OTU) assignment and analyses of community alpha and beta diversity were analyzed according to the Quantitative Insights Into Microbial Ecology (QIIME2) bioinformatics pipeline.[18] Quantitative polymerase chain reaction (qPCR) was performed on QuantStudio 12K Flex Real-Time PCR System (Applied Biosystems, Grand Island, NY). All qPCR experiments included appropriate negative (no DNA) and positive control reactions. No amplification was detected in

negative controls (Ct > total cycles). The analysis was conducted in triplicate to ensure the statistical validity of the results.

S5. Additional SBR operation results

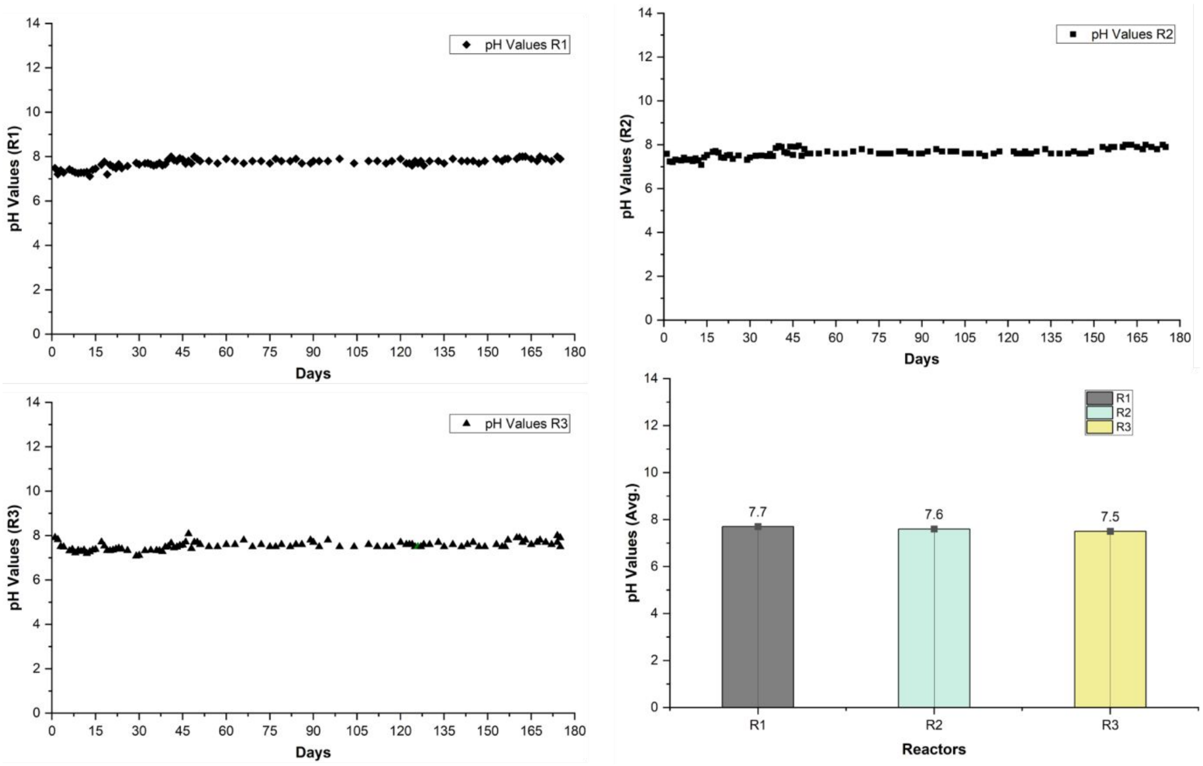

Figure S2. Effluent pH values of the reactors over the operation period for R1, R2, and R3

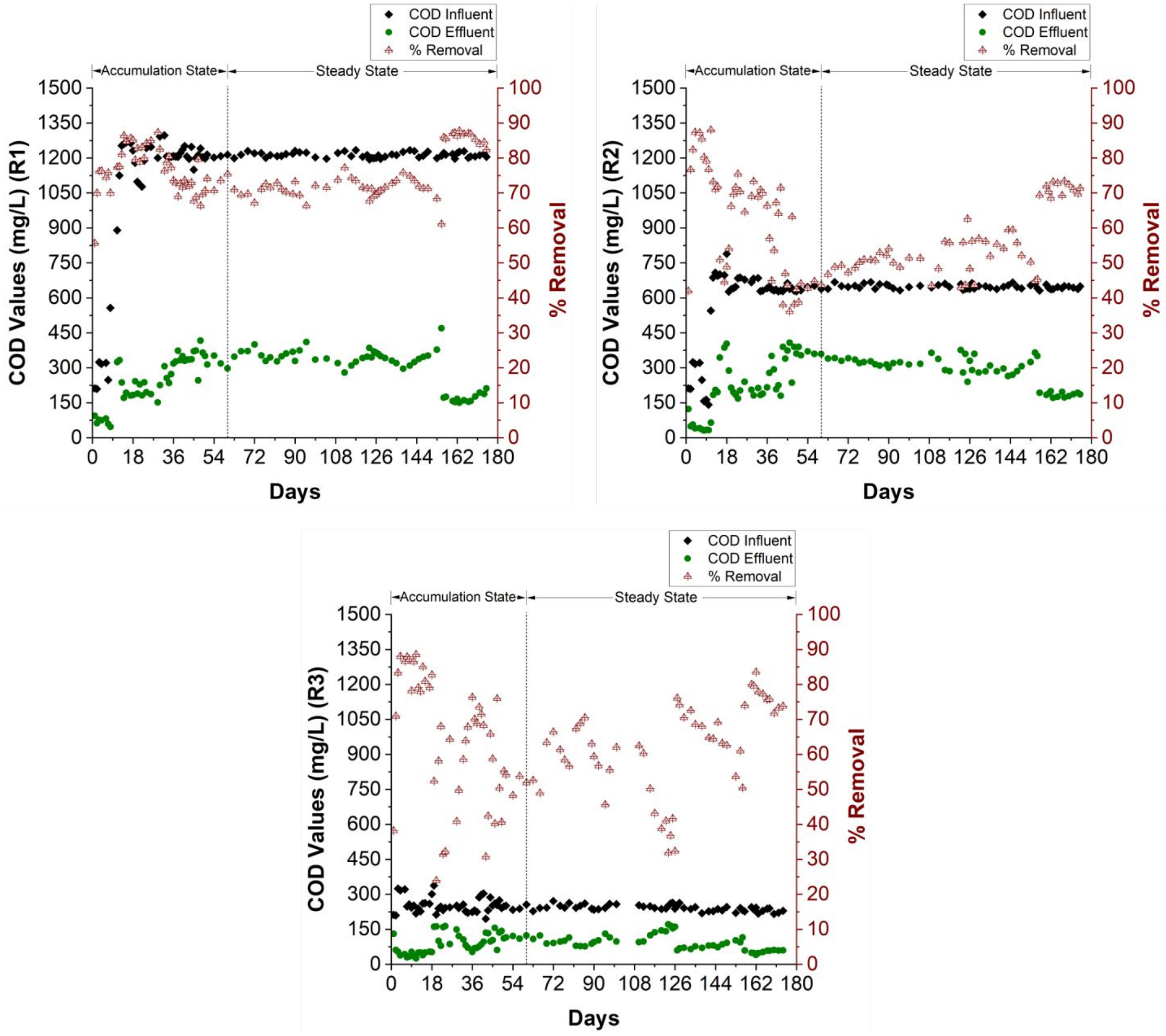

Figure S3. The influent and effluent COD values for R1, R2, and R3 reactors with respective removal efficiencies over the study period.

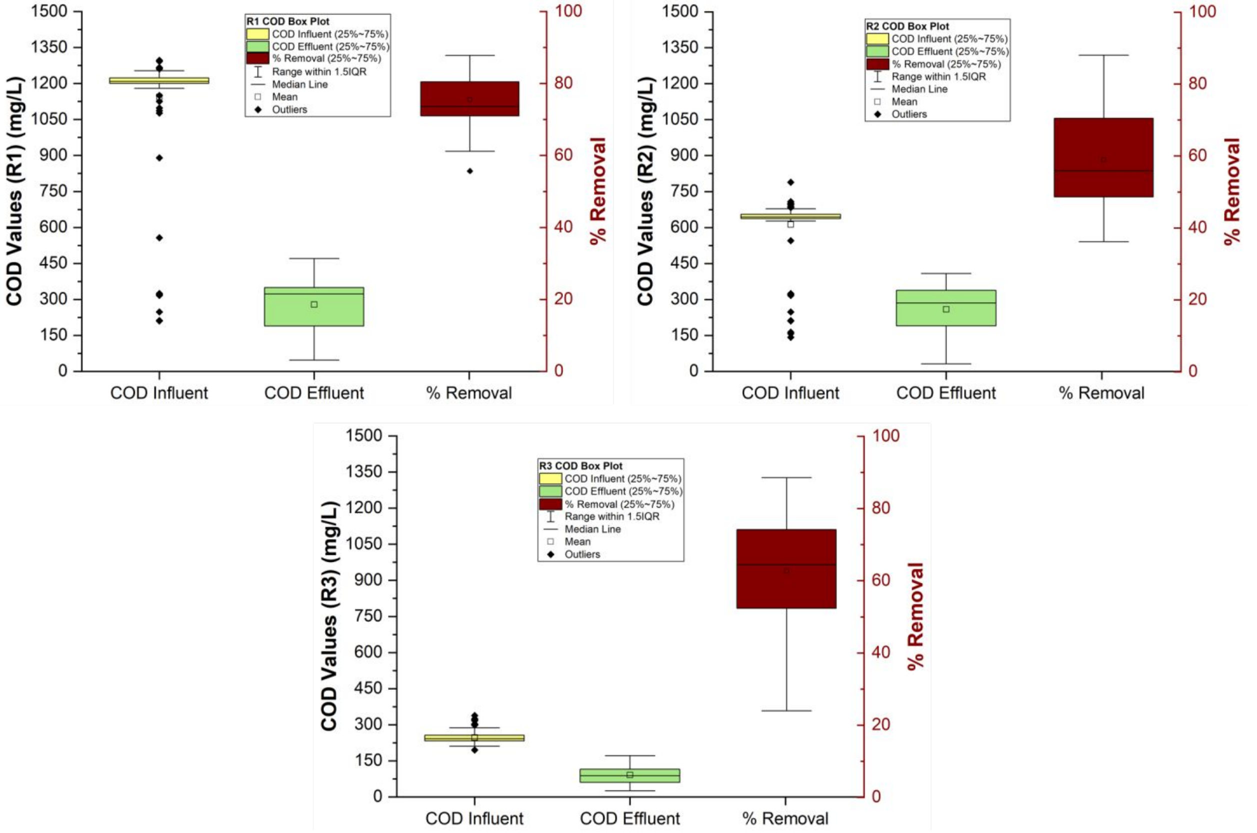

Figure S4. Boxplot of the influent and effluent COD values with removal efficiencies for R1, R2, and R3

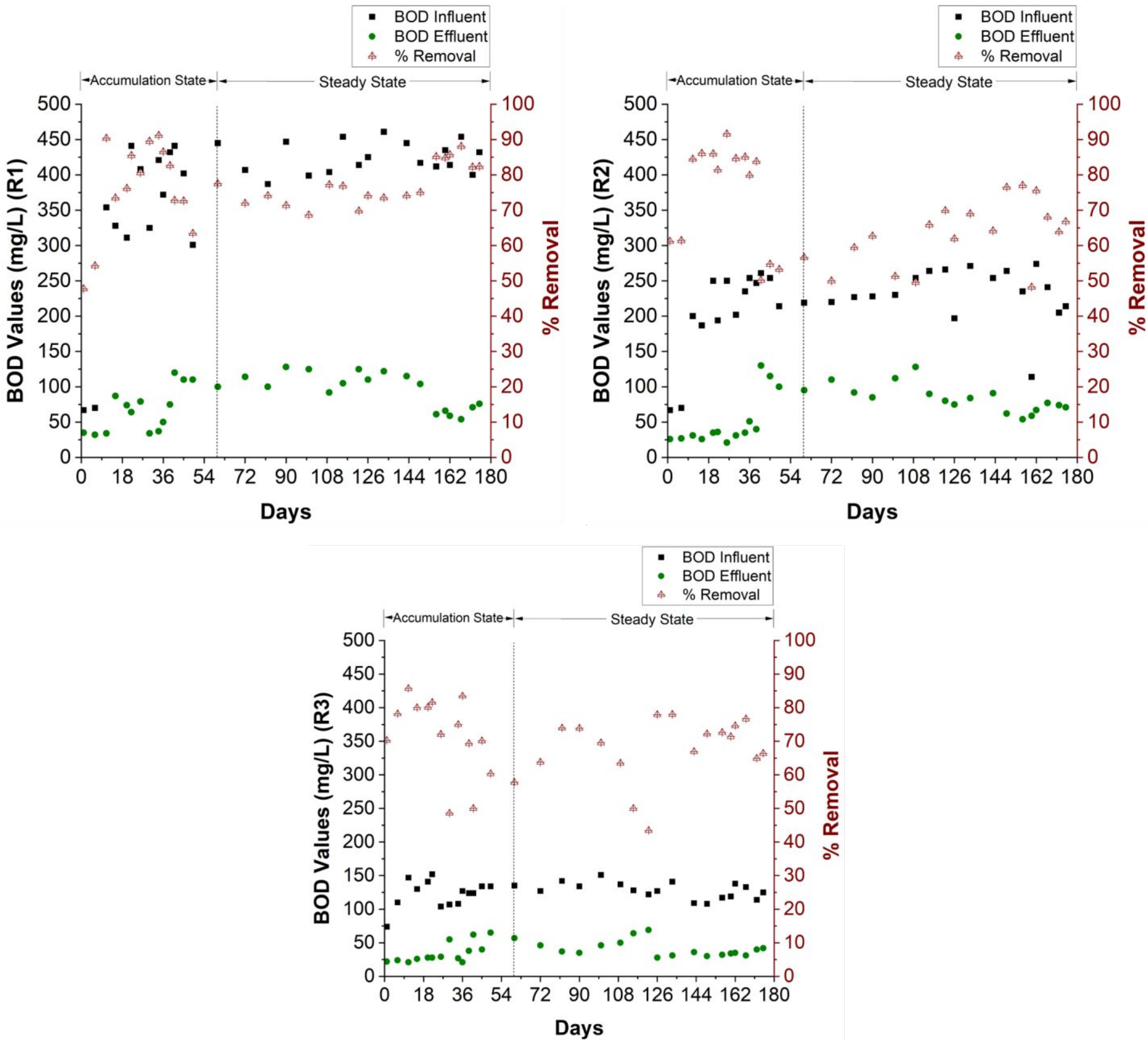

Figure S5. The influent and effluent BOD values for R1, R2, and R3 reactors with respective removal efficiencies over the study period.

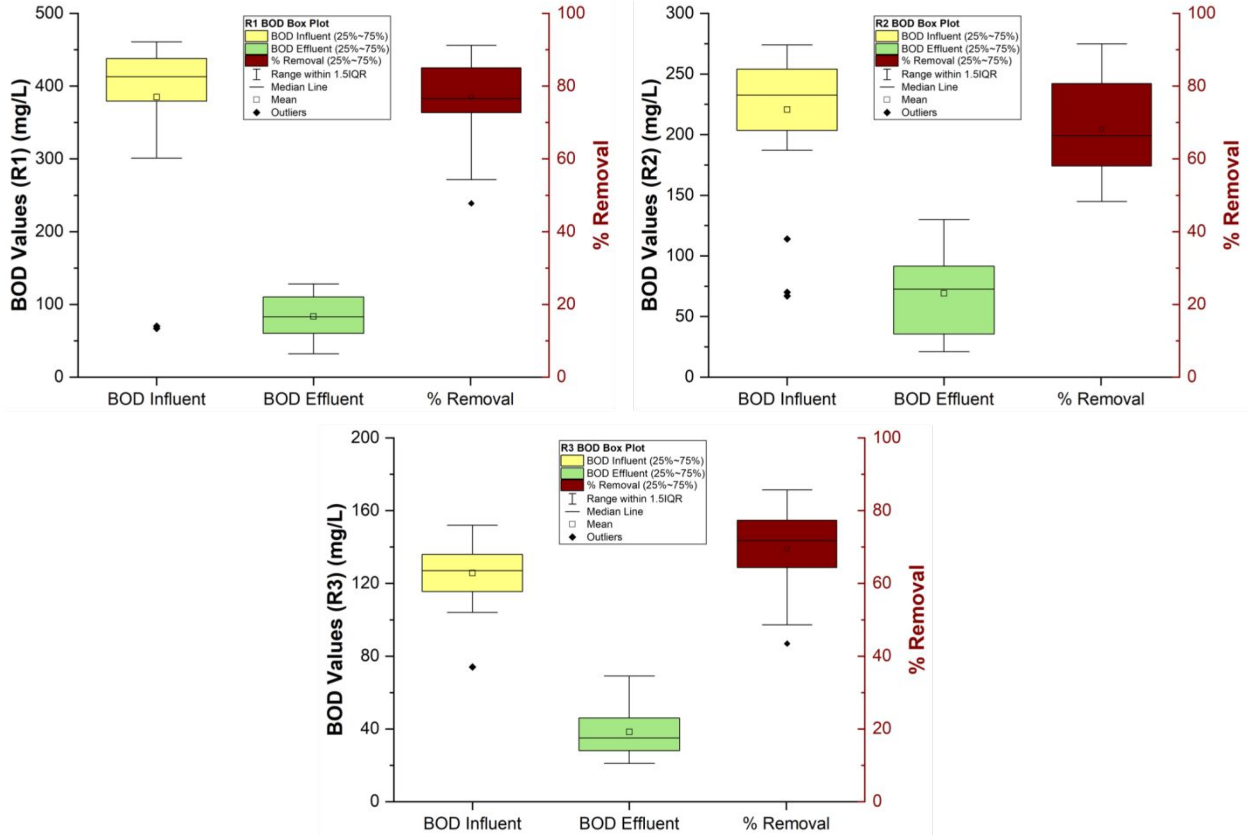

Figure S6. Boxplot of the influent and effluent BOD values with removal efficiencies for R1, R2, and R3.

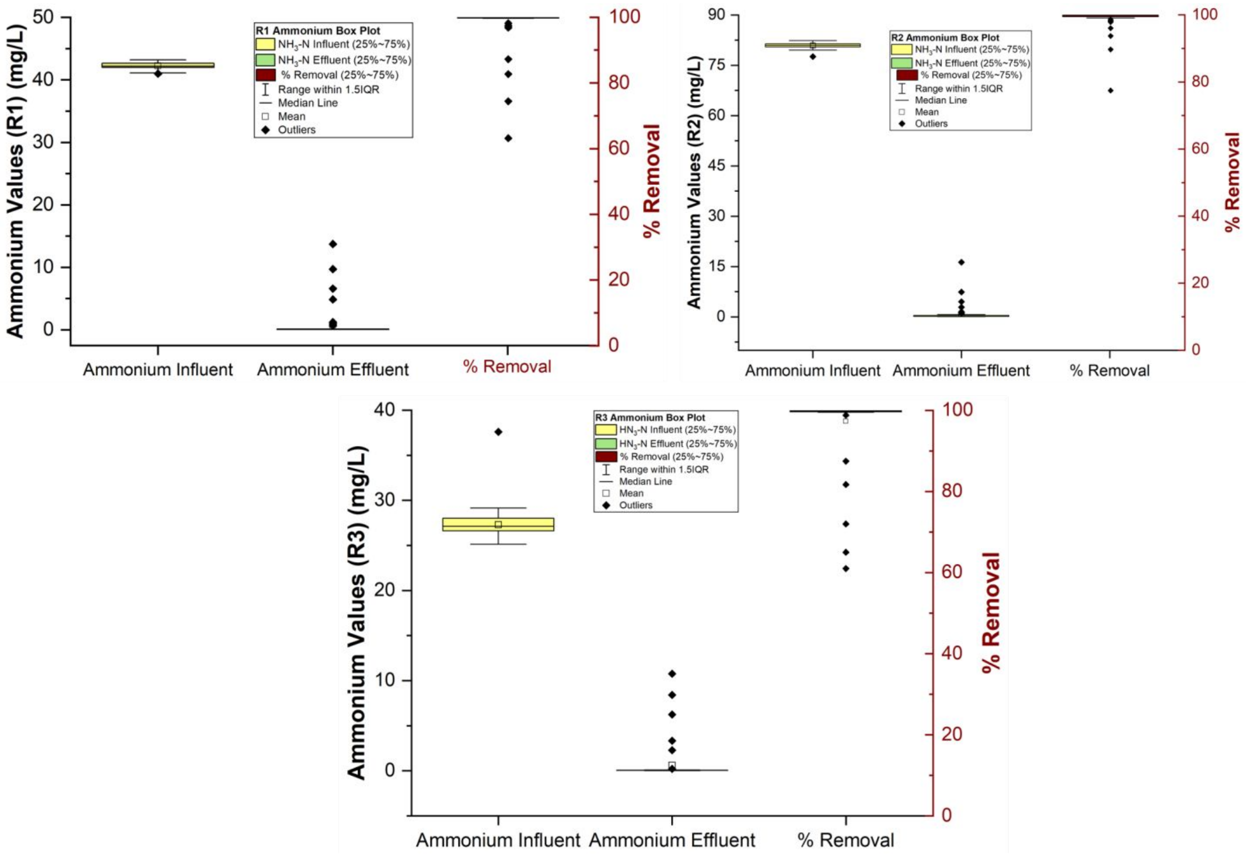

Figure S7. Boxplot of the influent and effluent ammonium values with removal efficiencies for R1, R2, and R3

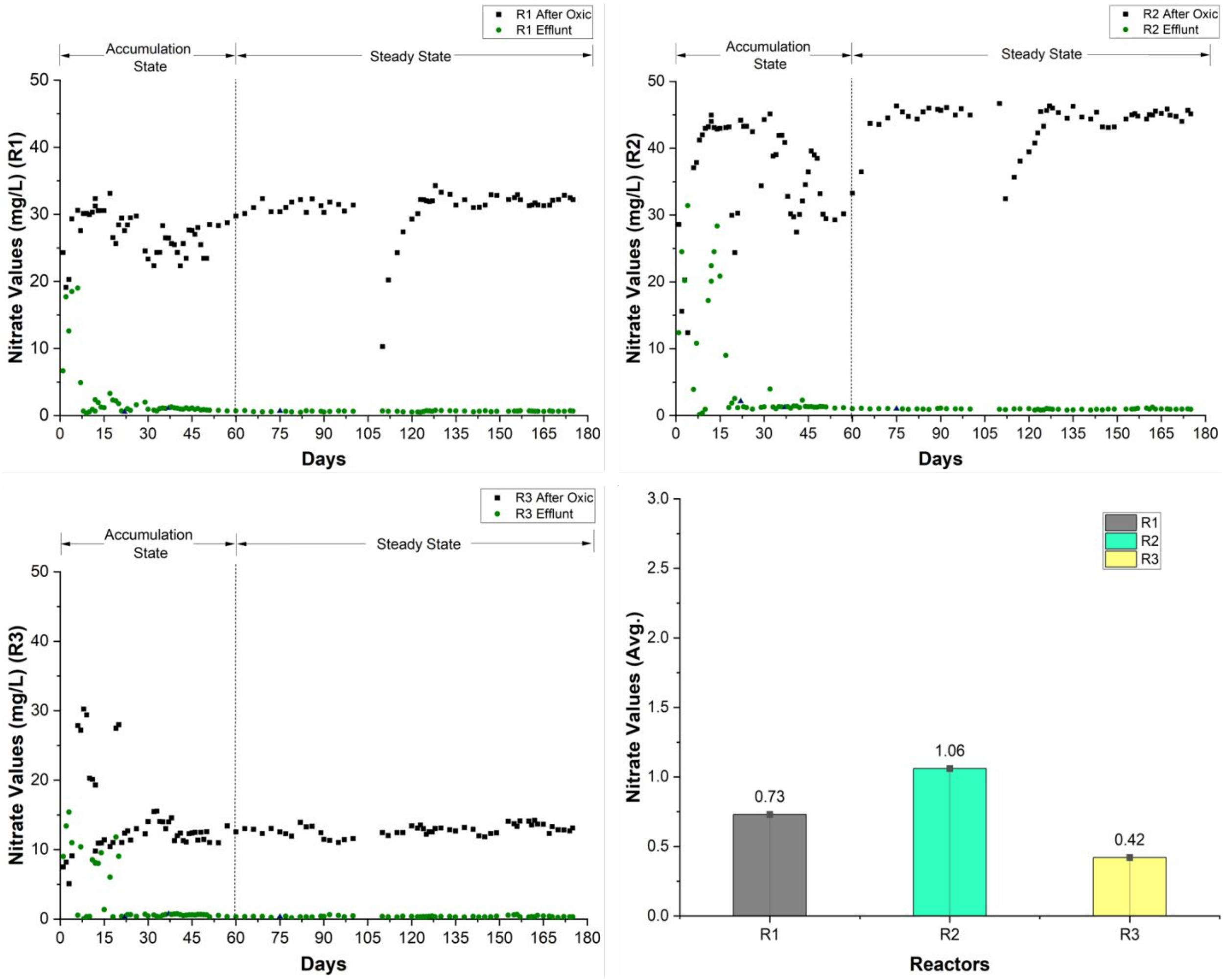

Figure S8. The post-nitrification (after oxic) and post-denitrification (effluent) nitrate values for R1, R2, and R3, along with the average effluent nitrate at the steady-state condition.

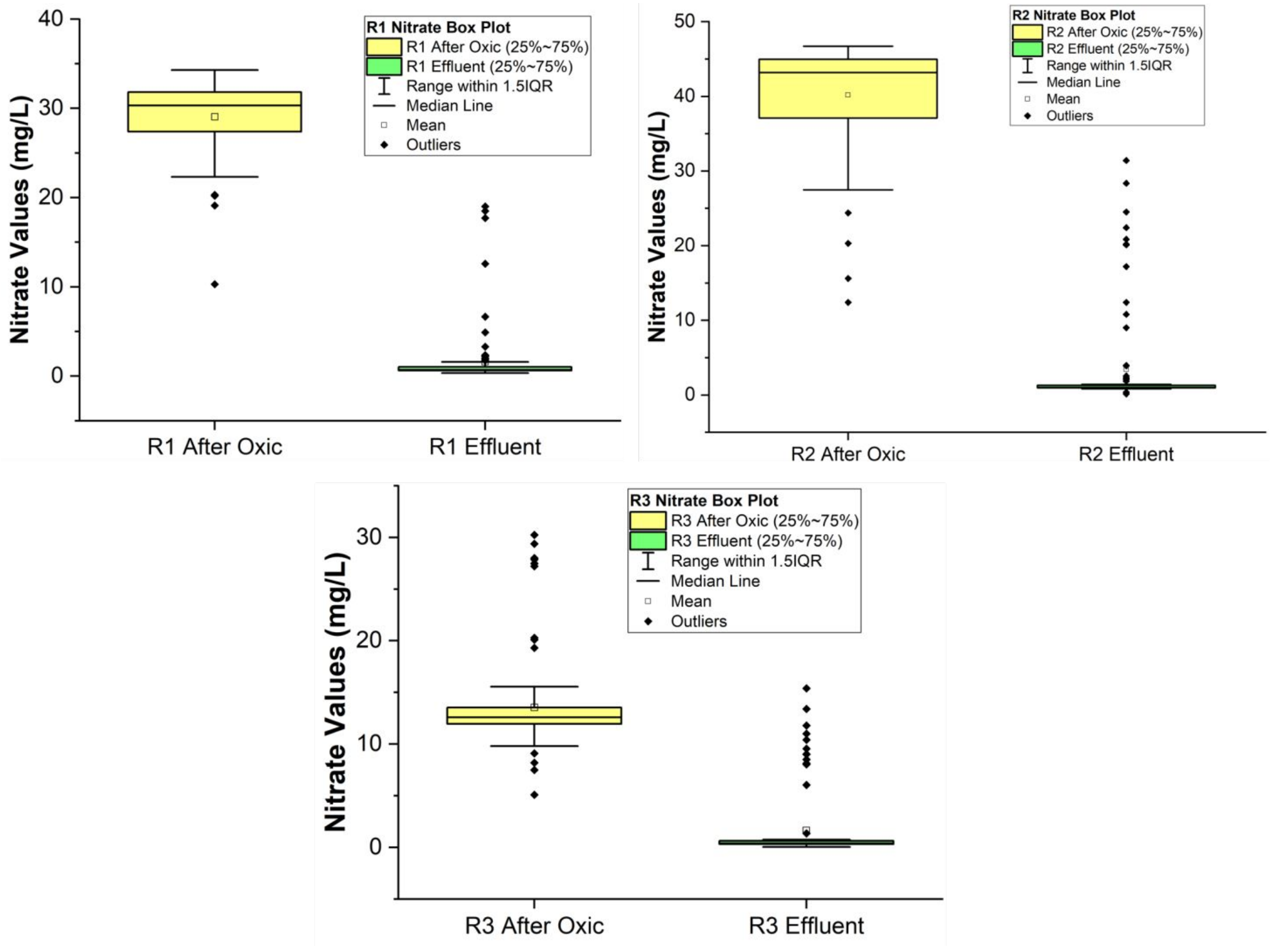

Figure S9. The box plot of post-nitrification (after oxic) and post-denitrification (effluent) nitrate values for R1, R2, and R3.

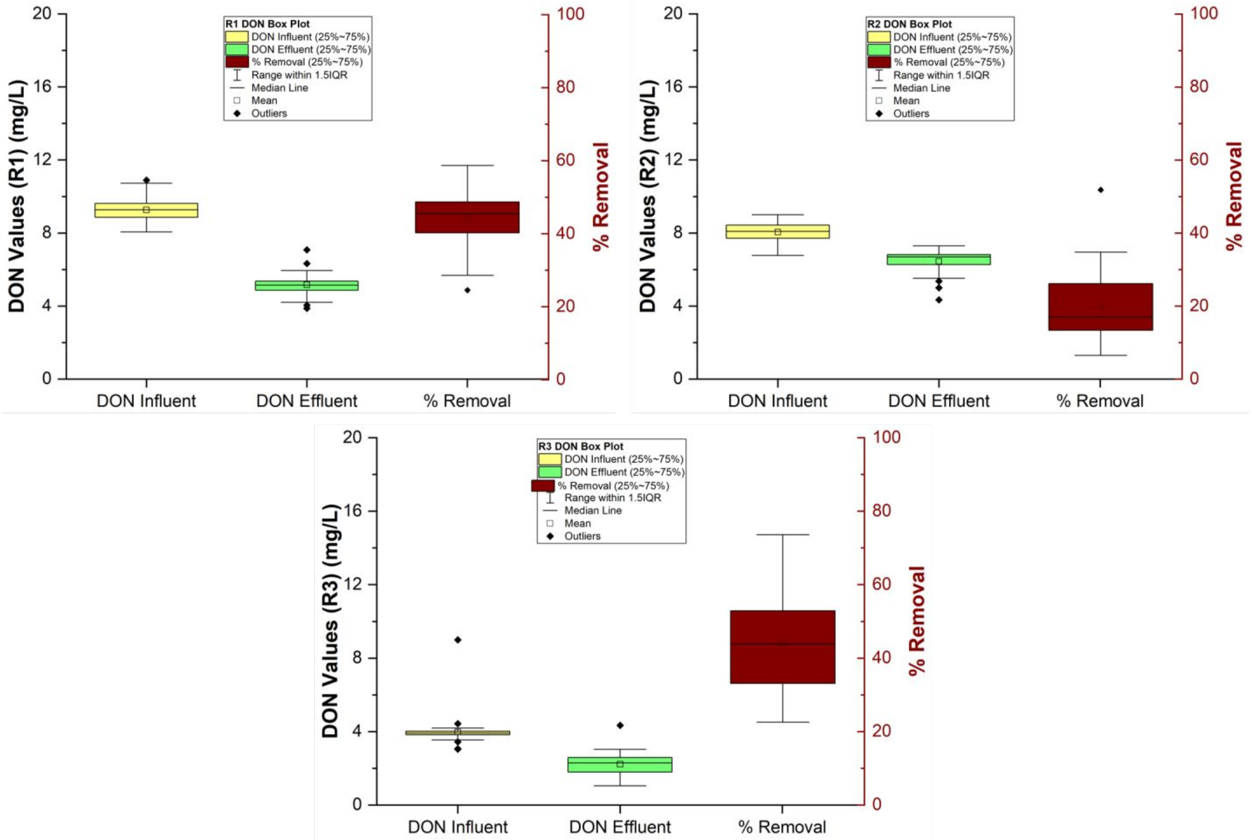

Figure S10. Boxplot of the influent and effluent DON values with removal efficiencies for R1, R2, and R3

S6. ^{13}C NMR spectroscopic analysis

NMR analyses reveal structures of organic components in samples, with the ^{13}C NMR spectrum segmented into eight zones corresponding to functional groups identified in DOM studies from rivers, sewage, and leachates [25–30]. The specified chemical shift regions (δ, ppm) include: I (0-46): Alkyl C, II (62-46): Methoxyl/N-substituted alkyl Carbons (OCH_3/NCH), III (62-46): O-alkyl C, IV (93-110): Anomeric C, V (110–145): Aromatic C, VI (145–161): Aromatic C–O (mainly from phenolic C), VII (161–191): Carbonyl C in carboxylic/ester/amide, and VIII (191–220): Carbonyl C in ketones/aldehydes [25–27,30,31]. The ^{13}C spectrum of Leachate A, B, and sewage showed multiple peaks, indicating DOM in various regions. Figure S12 shows the ^{13}C NMR spectrum, while Table S3 shows the chemical shift regions (δ, ppm) and their components.

Leachates and sewage showed two peaks at 10 and 13 ppm in the alkyl carbon region (Figure S12 (a) and (b) and (c)). Both leachates had a moderate peak at 19 ppm. Two prominent peaks at 30-31 and 23 ppm corresponded to methylene ($–CH_2$) and methyl ($–CH_3$) groups associated with

proteins and long fatty acid chains [32,33]. Leachates A and B showed mild peaks at 39 ppm and 37 ppm, likely due to quaternary carbon (Cq) or methylene carbon bound to nitrogen atoms (N–CH_2) [27,31]. Previous studies on landfill leachate-DOM have consistently reported a peak of around 37 ppm [25,27]. The 37 ppm peak found in leachate DOM was absent in various other studies [34]. Leachate B showed minor peaks at 44 and 61 ppm, and Leachate A had a prominent peak at 62 ppm, indicative of carbon in peptides, amino acids, [32,33] and methoxyl groups from lignin-like compounds [35–37]. Leachate A displayed a clear O-alkyl C peak at 71 ppm, signifying carbohydrates in the DOM, often identified as cellulose residues [35,38]. Moderate peaks at 129 and 138 ppm in Leachate B suggest oxygen/nitrogen-substituted aromatics, amines, lignins, phenols, or aromatic ethers ethers [32,33,36–38]. A moderate COO/N–C=O signal (a protein-like component) at 175 ppm in Leachate B's spectra was also found. Another study identified a peak at 173 ppm as the ester group from suberin and hemicellulose, carboxyl groups in suberin and/or lignin aromatic structures, and uronic acids.[37] Significant peaks at 162 ppm in sewage, and at 181 ppm, as well as between 184-185 ppm in both Leachate B and sewage, correspond to carbonyl C in carboxylic/ester/amide compounds [32,36,38,39]. No detectable signals between 191-220 ppm in leachates and sewage indicate minimal or no carbonyl C from ketones/aldehydes in the DOM.

260

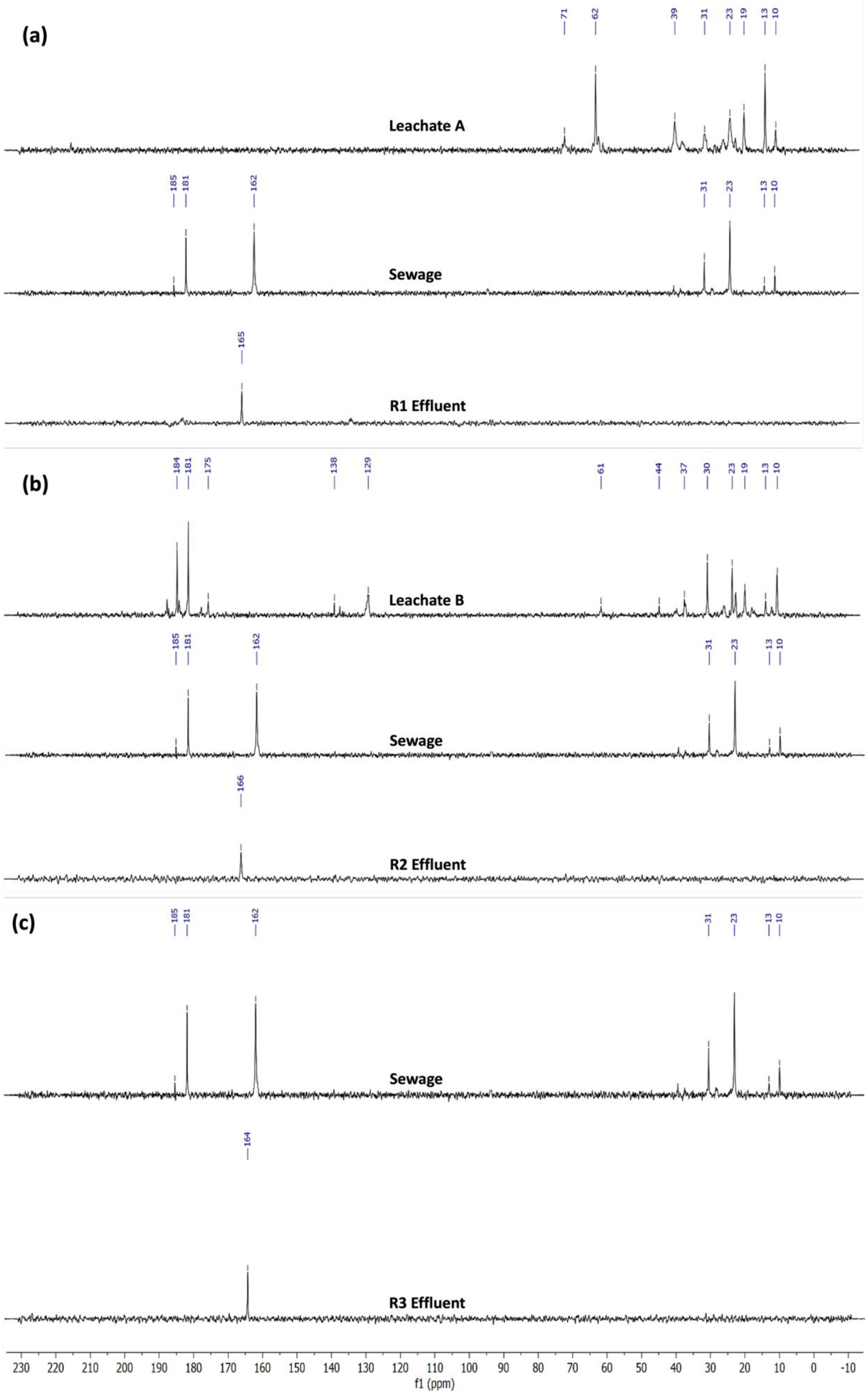

(a)
71
62
39
31
23
19
13
10
Leachate A
185
181
162
31
23
13
10
Sewage
165
R1 Effluent
(b)
184
181
175
138
129
61
44
37
30
23
19
13
10
Leachate B
185
181
162
31
23
13
10
Sewage
166
R2 Effluent
(c)
185
181
162
31
23
13
10
Sewage
164
R3 Effluent
230 220 210 200 190 180 170 160 150 140 130 120 110 100 90 80 70 60 50 40 30 20 10 0 -10
f1 (ppm)

Figure S11. ^{13}C NMR spectrum of the leachate and effluent samples: (a) Leachate A, sewage, and R1 effluent, (b) Leachate B, sewage, and R2 effluent, and (c) sewage and R3 effluent. 0.8% of Leachate A and 3% of Leachate B were introduced into the sewage as influent for R1 and R2, respectively.

Table S1. The detected chemical shift regions in the samples using ^{13}C NMR spectroscopy.

	**Samples**	**Leachate A**	**Leachate B**	**Sewage**	**R1 Effluent**	**R2 Effluent**	**R3 Effluent**
		**Chemical shift region δ (ppm)**					
**Assignment** [25–27,30,31]	Alkyl C	10	10	10			
	Alkyl C	13	13	13			
	Methyl groups	19	19				
	Alkyl C (protein-like component)	23	23	23			
	Alkyl C (protein-like component)	31	30	31			
	Quaternary C (Cq)/ methylene C bonded to N atoms ($N–CH_2$)	39	37				
	Carbon in peptides and amino acids (NCH)		44				
	Carbon in peptides and amino acids (NCH)	62	61				
	O-alkyl C	71					
	aromatic C peak		129				
	pyridine		138				
	Carbonyl C in carboxylic/ester/amide			162	165	166	164
	Carbonyl C peak (protein-like		175				

component) COO/N–C=O			
Carbonyl C in carboxylic/ester/amide	181	181	181
Carbonyl C in carboxylic/ester/amide	184	185	185

During biological treatment, all peaks were eliminated except for one at 164-166 ppm, which persisted across all reactors (R1, R2, R3). This indicates the resistance of Carbonyl C in carboxylic/ester/amide compounds to biological degradation [32,36,38,39]. NMR analysis revealed DOM complexity in leachate and sewage, which is critical for evaluating BNR treatment efficacy. Influent samples showed various functional groups, reflecting a wide range of nitrogenous compounds. Peaks at 37 ppm, 62 ppm, and 71 ppm indicated nitrogenous substances from proteins, amino acids, and carbohydrates in the leachates. Biological treatment largely succeeded in peak removal, but a persistent signal at 164-166 ppm underscored the difficulty of biologically degrading carbonyl carbons.

S7. FTIR spectroscopic analysis

Due to the proportional contributions of different functional groups to leachate chemistry, FTIR band ratios are a useful semi-quantitative indicator of organic matter quality changes [40,41]. Figure S12 shows FTIR spectra, while Table S2 lists DOM signals and functional groups. Table S2 and Figure S12 reveal two distinct bands in the FTIR spectra of leachate samples (L-A & L-B) and sewage, ranging from 3190-3350 to 2930 cm^{-1}. These bands represent primary amides/amines' NH stretch (II) and amine salts' N-H stretching. Lenz et al. (2016) found that NH_4-N concentration can be determined by analyzing the NH2 stretch, a broad band above 3000 cm^{-1}.[42] A spectral feature was observed in the sewage sample at 1640-1690 cm^{-1}, attributed to imine/oxime functional group C=N stretching. Further, FTIR spectra of leachate and sewage samples revealed two significant absorption bands at 1540-1560 and 1340-1400 cm^{-1}, indicating aliphatic nitro compounds. The noticeable NH in-plane vibration band at 1560 cm^{-1} indicates NH_4-N concentration [42,43]. The peak at 1400 cm^{-1} is caused by functional groups such as C-N bond stretching, N-H bond deformation, and C-H bond deformation [44]. Leachates and sewage have elevated ammonium and nitrate levels, confirming the bands. Similar results were reported by [45] and in their study of eight fractions of water-extractable organic matter (WEOM) utilizing FTIR spectroscopy. In contrast, both leachates showed a single deficient band at 1295 cm^{-1}. Small peaks at 1295 cm^{-1}, caused by CN stretching vibrations in amides, have been observed in leachate samples with high NH_4-N levels [42]. The presence of 1295 cm^{-1} was not detected in sewage due to its lower NH4-N concentration compared to leachates. The wavenumber range of 1080-1140/610-680 cm^{-1} was found in both leachates and sewage, suggesting a link to inorganic sulfate stretching. The crucial soluble aspects of landfill leachate, as noted in previous studies [46–48] are the functional groups associated with wavenumbers of 3,330, 2,900, 1,600, and 1,067 cm^{-1}. These bands consistently exhibit characteristic features across diverse types of leachates.

SBR effluents (R1 and R2) in leachate samples exhibit key spectral features in the ranges of 3380-3415 cm^{-1}, 1640-1690 cm^{-1}, 1400 cm^{-1}, 1140-1080 cm^{-1}, and 680-610 cm^{-1}. The effluent from R3's sewage follows a parallel pattern, but lacks the 3380-3415 cm^{-1} and 1400 cm^{-1} peaks and has an additional peak at 3460-3510 cm^{-1}. The peak at 700-750 cm^{-1} is shared by R2 and R3, but not by R1. Peaks at 3380-3415 cm^{-1} represent aliphatic primary amine NH stretch, 1640-1690 cm^{-1} represent imine/oxime functional group C=N stretching, 1400 cm^{-1} represent nitrate/nitrite ions, and 1140-1080 and 680-610 cm^{-1} represent inorganic sulfate S-O stretch. The peak at 3460-3510

cm⁻¹ represents the NH stretch of aromatic primary amine, while the peak at 700-750 cm⁻¹ represents the N-H in-plane vibration of amine/amide. This comprehensive spectral analysis reveals compositional differences in SBR effluents from different reactors, revealing chemical constituents and transformations in leachates and sewage samples.

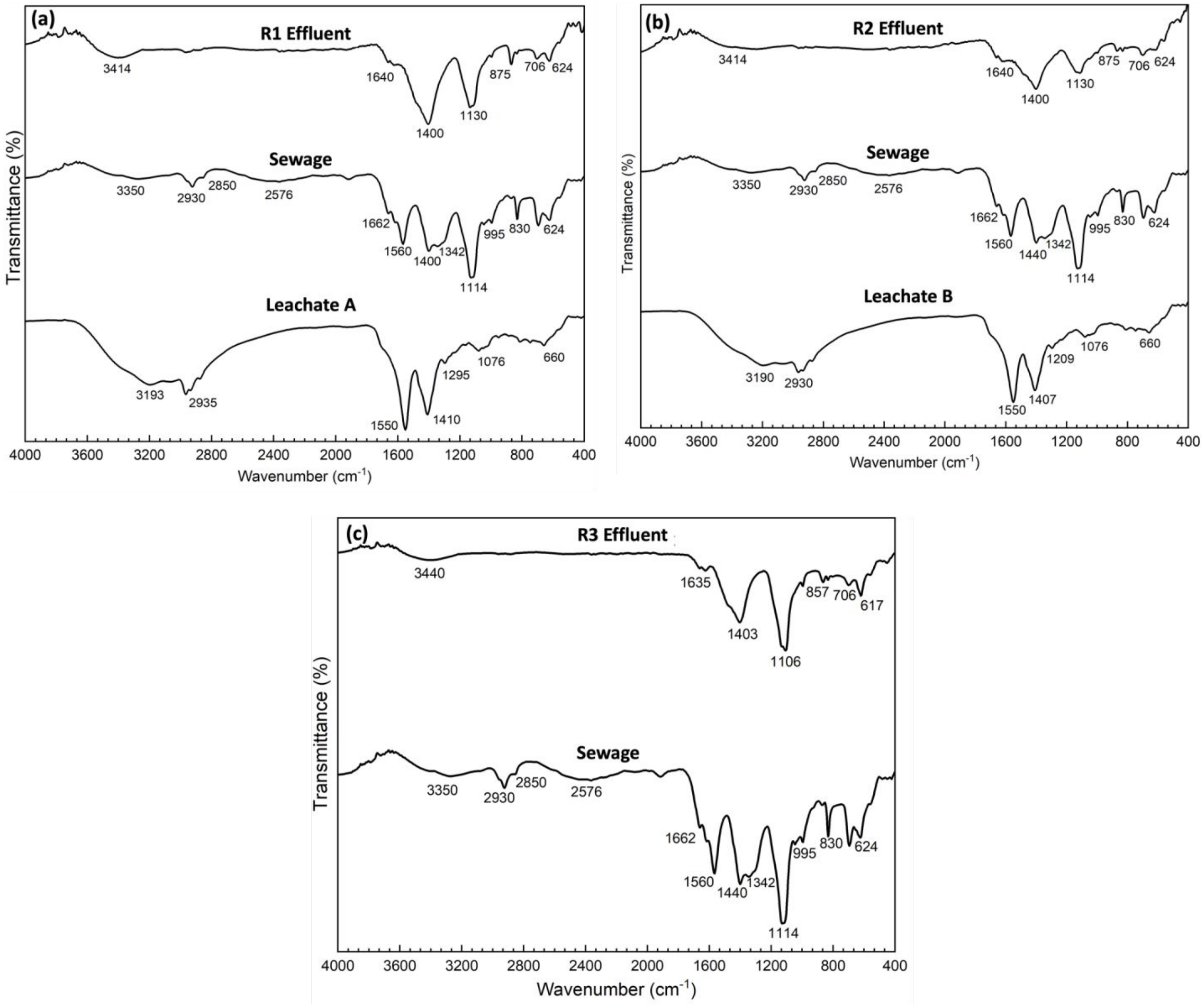

Figure S12. FTIR spectrum of the leachate and effluent samples: (a) Leachate A, sewage, R1 effluent, (b) Leachate B, sewage, and R2 effluent, and (c) sewage and R3 effluent. 0.8% of Leachate A and 3% of Leachate B were introduced into the sewage as influent for R1 and R2, respectively

Table S2. The FTIR signal of the samples with the relative functional group.

Functional Groups [49,50]	Samples with relative signals (cm^{-1})					
	Leachate A	Leachate B	Sewage	R1-Eff	R2-Eff	R3-Eff
Aromatic primary amine, NH stretch 3460-3510						3460
Aliphatic primary amine, NH stretch 3380-3415				3414	3415	
Aliphatic secondary amine, >N-H stretch 3310-3360			3350			
>3000 NH stretch (2 bands) Primary amides	3193	3190				
3000-2800 N-H stretching amine salt	2935	2930	2930			
1690-1640 C=N stretching imine / oxime			1662			
Organic nitrates 1620-1640				1640	1627	1635
Aliphatic nitro compounds 1540-1560	1550	1550	1560			
Nitrate/Nitrite ion (1330-1400)	1400	1400	1342	1400	1400	
C-N stretching of amides (1295)	1295	1295				
(1140–1080)/(680-610) S-O stretch Inorganic sulfates	1080/660	1080/660	1114/624	1130	1118	1106
850–750 NH2 stretch Primary amine group			830			
N-H in plane of amine/amide (700-750)					706	706

FTIR spectra show significant differences between influent and effluent samples in SBR. Influent samples contain ammonium indicators, nitrate/nitrite ions, aliphatic nitro compounds, amine salt N-H stretching, and functional groups. Reactor-specific patterns in effluent samples show either the removal of NH stretch (II) of primary amides/amines, N-H stretching of amine salt, aliphatic

nitro compounds, and CN stretching vibrations in amides or the transformation of them to NH stretch of aliphatic or aromatic primary amine, organic nitrates, and N-H in-plane vibration of amine/amide through the BNR (These distinctions highlight reactor-specific transformations, revealing SBR processes' leachate and sewage treatment efficacy.

S8. Additional microbial community analysis results

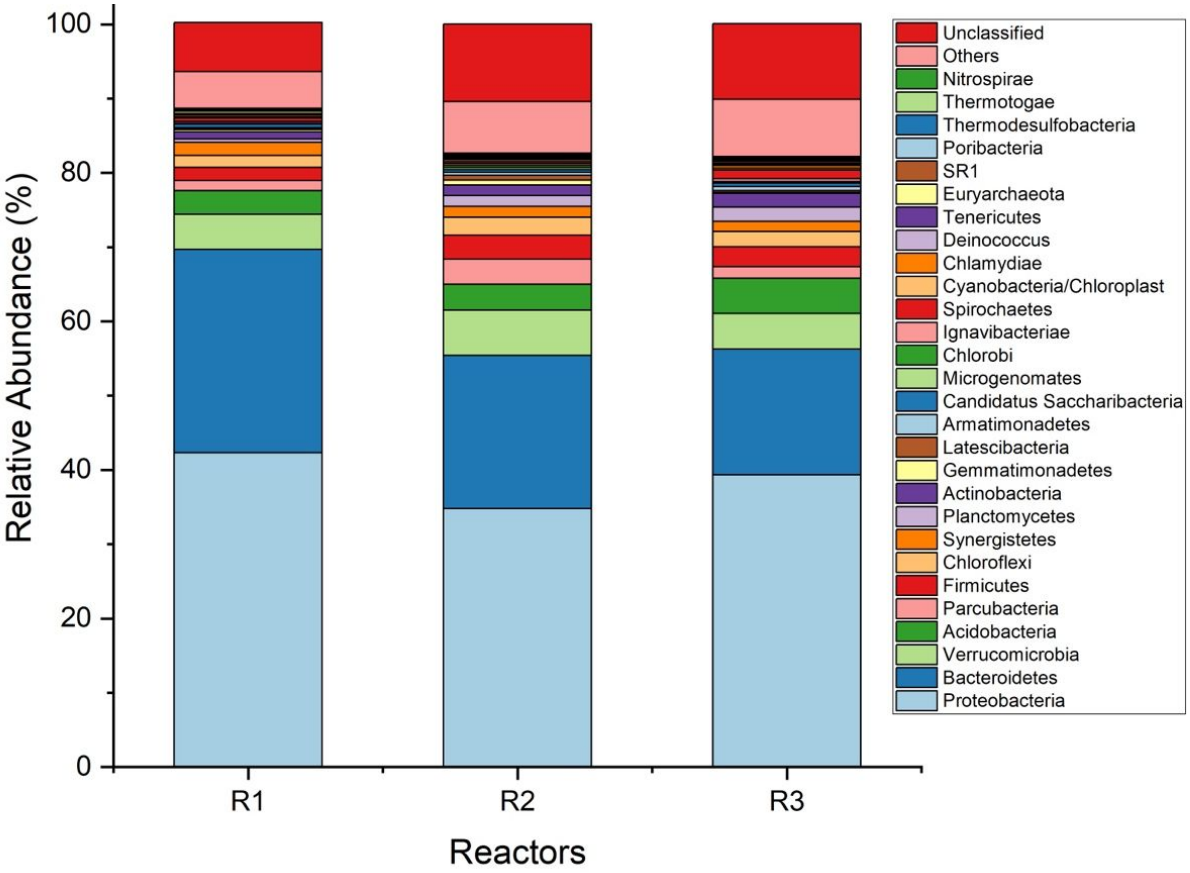

Figure S13. Phylum-level composition and relative abundance of activated sludge samples from R1, R2, and R3 reactors.

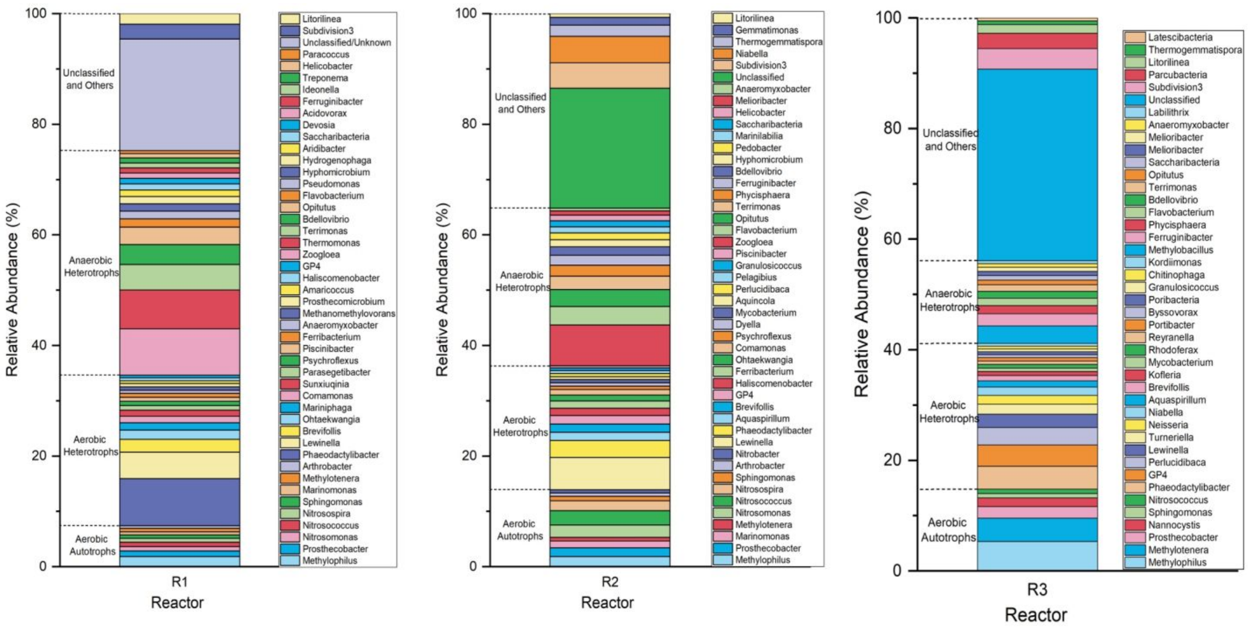

Figure S14. The genus level and relative abundance of the activated sludge samples from R1, R2, and R3.

S9. Additional *in-situ* algal bioassay results

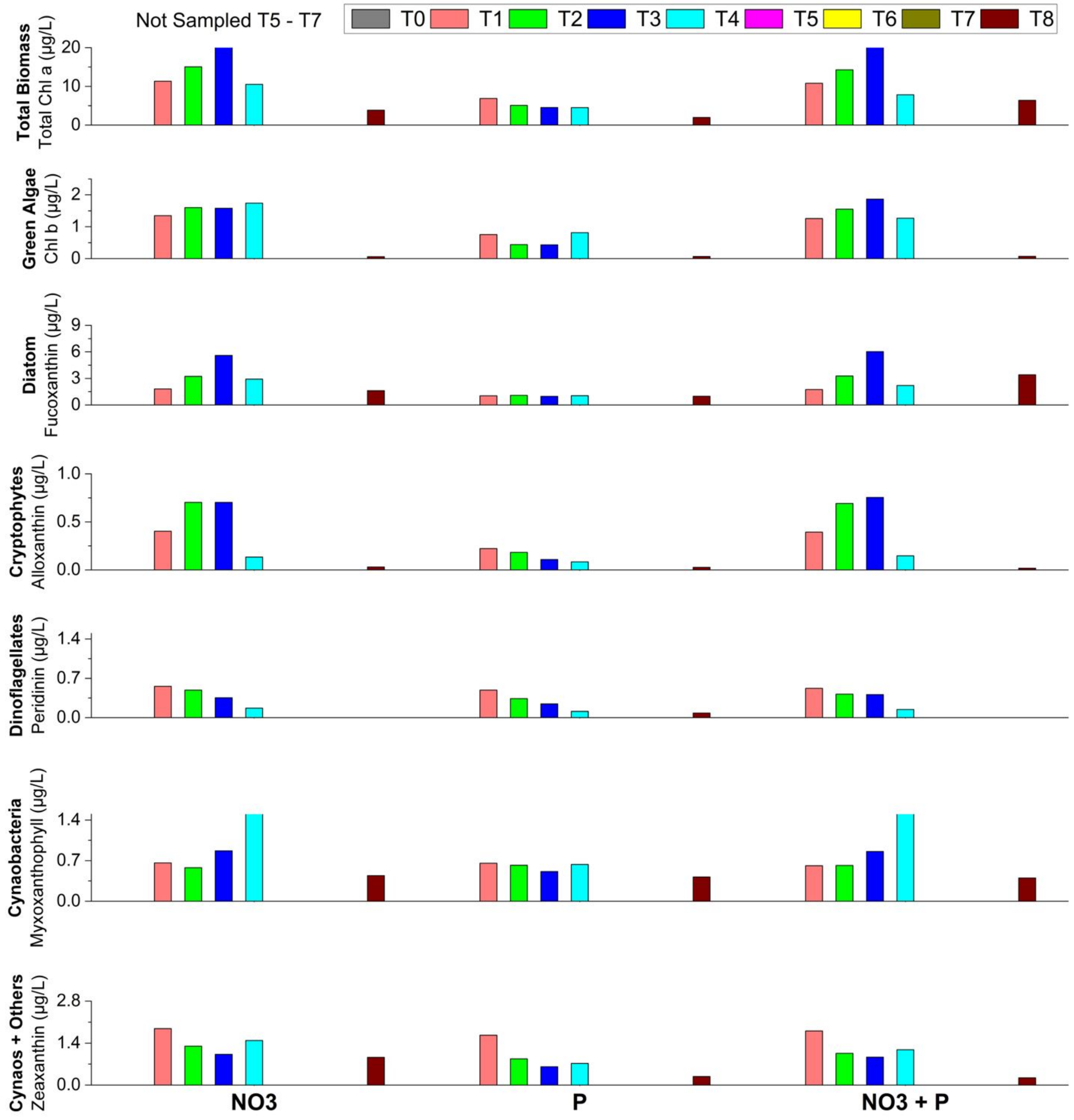

Figure S15. Algal group-spcific growth responses to nutrient (NO_3, P, and NO_3+P) spiked vs. control samples.

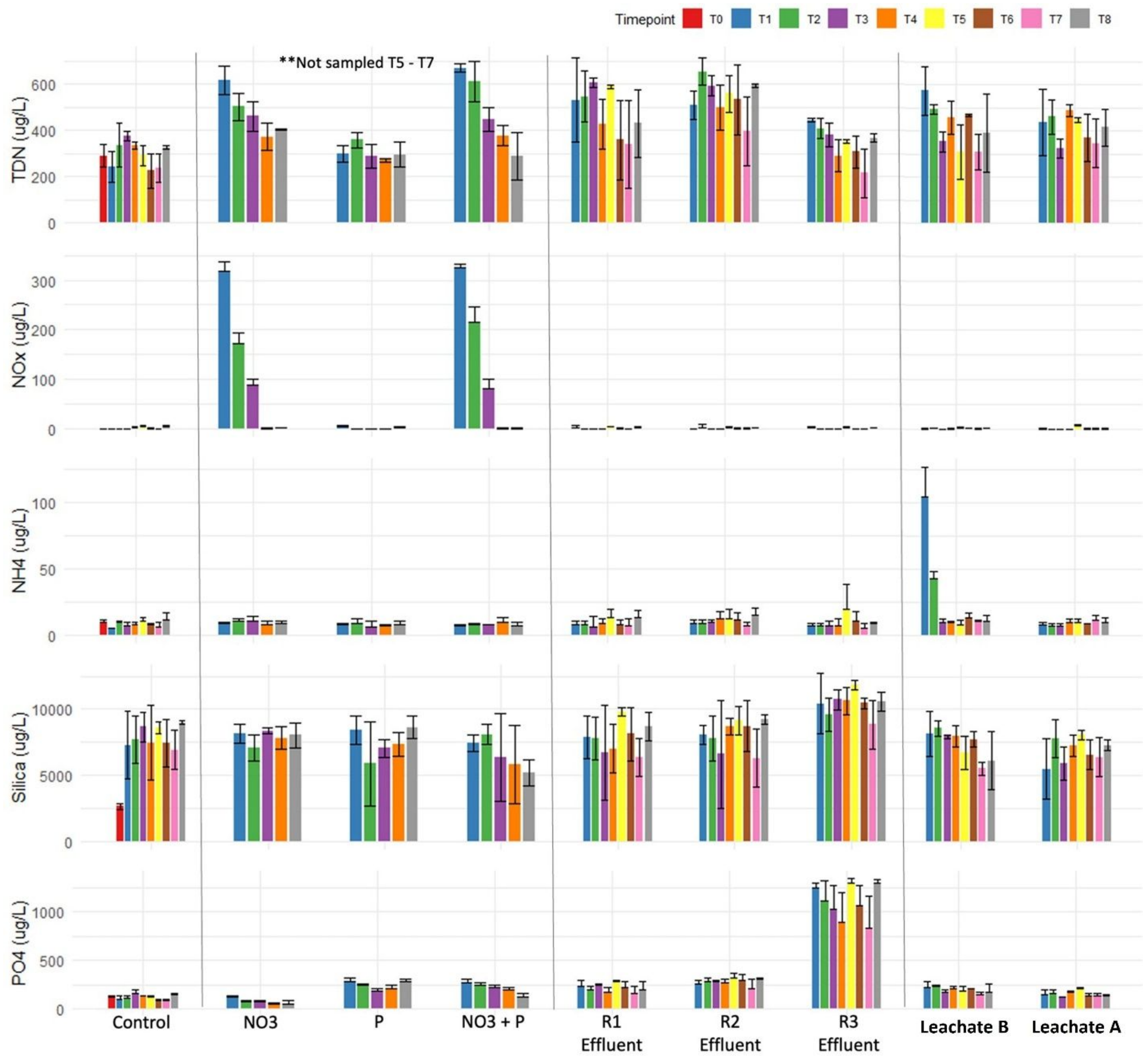

Figure S16. The nutrient data from T0 to T8 for the bioassay. (T0- day 1, T1- day 2, T2 - day 3, T3 - day 4, T4 - day 5, T5- day 8, T6- day 9, T7 - day 10, T8- day 11)

S10. DON utilization rate by algae

Table S3: Change in the DON concentration during the bioassay experiment

	**DON (µg/L)**								
**Sample**	Day 1	Day 2	Day 3	Day 4	Day 5	Day 8	Day 9	Day 10	Day 11
	To	T1	T2	T3	T4	T5	T6	T7	T8

R1 Effluent	499	499	520	590	390	558	329	310	389
R2 Effluent	490	490	629	570	470	519	505	290	465
R3 Effluent	414	414	385	355	275	309	285	200	345

DON utilization rate by algae (μg/L/day): Rate = $(C_t - C_0)/\Delta t$,

Here, C_t = DON concentration at Day 11 (μg/L)

C_0 = DON concentration at Day 1 (μg/L)

Δt = change in the time (days)

DON utilization rate by algea for R1 effluent = (499-389)/10 = 11 μg/L/day

DON utilization rate by algae for R2 effluent = (490-465)/10 = 2.5 μg/L/day

DON utilization rate by algae for R3 effluent = (414-345)/10 = 6.9 μg/L/day

S11. Statistical analysis of denitrification improvement

Minitab was used for the statistical analysis. and the p-value for the denitrification performance after methanol addition is 0.006. Below are the details:

Paired T-Test and CI: Before, After

Paired T for Before - After

	N	Mean	StDev	SE Mean
Before	3	25.00	10.22	5.90
After	3	97.40	0.36	0.21

Difference 3 -72.40 9.87 5.70

95% CI for mean difference: (-96.91, -47.89)

T-Test of mean difference = 0 (vs ≠ 0): T-Value = -12.71 P-Value = 0.006

References

(1) Emmett, M. R.; White, F. M.; Hendrickson, C. L.; Shi, S. D.-H.; Marshall, A. G. Application of Micro-Electrospray Liquid Chromatography Techniques to FT-ICR MS to Enable High-Sensitivity Biological Analysis. *J. Am. Soc. Mass Spectrom.* **1998**, *9* (4), 333–340. https://doi.org/10.1016/S1044-0305(97)00287-0.

(2) Hendrickson, C. L.; Quinn, J. P.; Kaiser, N. K.; Smith, D. F.; Blakney, G. T.; Chen, T.; Marshall, A. G.; Weisbrod, C. R.; Beu, S. C. 21 Tesla Fourier Transform Ion Cyclotron Resonance Mass Spectrometer: A National Resource for Ultrahigh Resolution Mass Analysis. *J Am Soc Mass Spectrom* **2015**, *26* (9), 1626–1632. https://doi.org/10.1007/s13361-015-1182-2.

(3) Smith, D. F.; Podgorski, D. C.; Rodgers, R. P.; Blakney, G. T.; Hendrickson, C. L. 21 Tesla FT-ICR Mass Spectrometer for Ultrahigh-Resolution Analysis of Complex Organic Mixtures. *Anal. Chem.* **2018**, *90* (3), 2041–2047. https://doi.org/10.1021/acs.analchem.7b04159.

(4) Kaiser, N. K.; McKenna, A. M.; Savory, J. J.; Hendrickson, C. L.; Marshall, A. G. Tailored Ion Radius Distribution for Increased Dynamic Range in FT-ICR Mass Analysis of Complex Mixtures. *Anal. Chem.* **2013**, *85* (1), 265–272. https://doi.org/10.1021/ac302678v.

(5) Chen, T.; Beu, S. C.; Kaiser, N. K.; Hendrickson, C. L. Note: Optimized Circuit for Excitation and Detection with One Pair of Electrodes for Improved Fourier Transform Ion Cyclotron Resonance Mass Spectrometry. *Rev Sci Instrum* **2014**, *85* (6), 066107. https://doi.org/10.1063/1.4883179.

(6) Boldin, I. A.; Nikolaev, E. N. Fourier Transform Ion Cyclotron Resonance Cell with Dynamic Harmonization of the Electric Field in the Whole Volume by Shaping of the Excitation and Detection Electrode Assembly. *Rapid Communications in Mass Spectrometry* **2011**, *25* (1), 122–126. https://doi.org/10.1002/rcm.4838.

(7) Blakney, G. T.; Hendrickson, C. L.; Marshall, A. G. Predator Data Station: A Fast Data Acquisition System for Advanced FT-ICR MS Experiments. *International Journal of Mass Spectrometry* **2011**, *306* (2), 246–252. https://doi.org/10.1016/j.ijms.2011.03.009.

(8) Xian, F.; Hendrickson, C. L.; Blakney, G. T.; Beu, S. C.; Marshall, A. G. Automated Broadband Phase Correction of Fourier Transform Ion Cyclotron Resonance Mass Spectra. *Anal. Chem.* **2010**, *82* (21), 8807–8812. https://doi.org/10.1021/ac101091w.

(9) Savory, J. J.; Kaiser, N. K.; McKenna, A. M.; Xian, F.; Blakney, G. T.; Rodgers, R. P.; Hendrickson, C. L.; Marshall, A. G. Parts-Per-Billion Fourier Transform Ion Cyclotron Resonance Mass Measurement Accuracy with a “Walking” Calibration Equation. *Anal. Chem.* **2011**, *83* (5), 1732–1736. https://doi.org/10.1021/ac102943z.
(10) Kendrick, Edward. A Mass Scale Based on CH2 = 14.0000 for High Resolution Mass Spectrometry of Organic Compounds. *Anal. Chem.* **1963**, *35* (13), 2146–2154. https://doi.org/10.1021/ac60206a048.
(11) Hughey, C. A.; Hendrickson, C. L.; Rodgers, R. P.; Marshall, A. G.; Qian, K. Kendrick Mass Defect Spectrum: A Compact Visual Analysis for Ultrahigh-Resolution Broadband Mass Spectra. *Anal. Chem.* **2001**, *73* (19), 4676–4681. https://doi.org/10.1021/ac010560w.
(12) Corilo, Y. E. PetroOrg Software, 2014.
(13) Paerl, H. W.; Bowles, N. D. Dilution Bioassays: Their Application to Assessments of Nutrient Limitation In. *Hydrobiologia* **1987**, *146* (3), 265–273. https://doi.org/10.1007/BF00016348.
(14) Paerl, H. W.; Xu, H.; Hall, N. S.; Rossignol, K. L.; Joyner, A. R.; Zhu, G.; Qin, B. Nutrient Limitation Dynamics Examined on a Multi-Annual Scale in Lake Taihu, China: Implications for Controlling Eutrophication and Harmful Algal Blooms. *Journal of Freshwater Ecology* **2015**, *30* (1), 5–24. https://doi.org/10.1080/02705060.2014.994047.
(15) Paerl, H. W.; Rossignol, K. L.; Hall, S. N.; Peierls, B. L.; Wetz, M. S. Phytoplankton Community Indicators of Short- and Long-Term Ecological Change in the Anthropogenically and Climatically Impacted Neuse River Estuary, North Carolina, USA. *Estuaries and Coasts* **2010**, *33* (2), 485–497. https://doi.org/10.1007/s12237-009-9137-0.
(16) Pinckney, J. L.; Richardson, T. L.; Millie, D. F.; Paerl, H. W. Application of Photopigment Biomarkers for Quantifying Microalgal Community Composition and in Situ Growth Rates. *Organic Geochemistry* **2001**, *32* (4), 585–595. https://doi.org/10.1016/S0146-6380(00)00196-0.
(17) Wang, Q.; Garrity, G. M.; Tiedje, J. M.; Cole, J. R. Naïve Bayesian Classifier for Rapid Assignment of rRNA Sequences into the New Bacterial Taxonomy. *Applied and Environmental Microbiology* **2007**, *73* (16), 5261–5267. https://doi.org/10.1128/AEM.00062-07.
(18) Caporaso, J. G.; Kuczynski, J.; Stombaugh, J.; Bittinger, K.; Bushman, F. D.; Costello, E. K.; Fierer, N.; Peña, A. G.; Goodrich, J. K.; Gordon, J. I.; Huttley, G. A.; Kelley, S. T.; Knights, D.; Koenig, J. E.; Ley, R. E.; Lozupone, C. A.; McDonald, D.; Muegge, B. D.; Pirrung, M.; Reeder, J.; Sevinsky, J. R.; Turnbaugh, P. J.; Walters, W. A.; Widmann, J.; Yatsunenko, T.; Zaneveld, J.; Knight, R. QIIME Allows Analysis of High-Throughput Community Sequencing Data. *Nat Methods* **2010**, *7* (5), 335–336. https://doi.org/10.1038/nmeth.f.303.
(19) Deng, Y.; Jung, C.; Zhao, R.; Torrens, K.; Wu, L. Adsorption of UV-Quenching Substances (UVQS) from Landfill Leachate with Activated Carbon. *Chemical Engineering Journal* **2018**, *350*, 739–746. https://doi.org/10.1016/j.cej.2018.04.056.
(20) Murphy, K. R.; Hambly, A.; Singh, S.; Henderson, R. K.; Baker, A.; Stuetz, R.; Khan, S. J. Organic Matter Fluorescence in Municipal Water Recycling Schemes: Toward a Unified PARAFAC Model. *Environ. Sci. Technol.* **2011**, *45* (7), 2909–2916. https://doi.org/10.1021/es103015e.
(21) Cory, R. M.; Kaplan, L. A. Biological Lability of Streamwater Fluorescent Dissolved Organic Matter. *Limnology and Oceanography* **2012**, *57* (5), 1347–1360.

(22) Xiao, K.; Shen, Y.; Liang, S.; Tan, J.; Wang, X.; Liang, P.; Huang, X. Characteristic Regions of the Fluorescence Excitation–Emission Matrix (EEM) To Identify Hydrophobic/Hydrophilic Contents of Organic Matter in Membrane Bioreactors. *Environ. Sci. Technol.* **2018**, *52* (19), 11251–11258. https://doi.org/10.1021/acs.est.8b02684.

(23) Carstea, E. M.; Bridgeman, J.; Baker, A.; Reynolds, D. M. Fluorescence Spectroscopy for Wastewater Monitoring: A Review. *Water Research* **2016**, *95*, 205–219. https://doi.org/10.1016/j.watres.2016.03.021.

(24) Dubber, D.; Gill, L. W. Suitability of Fluorescent Whitening Compounds (FWCs) as Indicators of Human Faecal Contamination from Septic Tanks in Rural Catchments. *Water Research* **2017**, *127*, 104–117. https://doi.org/10.1016/j.watres.2017.10.005.

(25) Chai, M. N.; Isa, M. I. N. The Oleic Acid Composition Effect on the Carboxymethyl Cellulose Based Biopolymer Electrolyte. **2013**, *2013*. https://doi.org/10.4236/jcpt.2013.31001.

(26) Dignac, M.-F.; Ginestel, P.; Bruchet, A.; Audic, J.-M.; Derenne, S.; Largeau, C. Changes in the Organic Composition of Wastewater during Biological Treatment as Studied by NMR and IR Spectroscopies. *Water Science and Technology* **2001**, *43* (2), 51–58. https://doi.org/10.2166/wst.2001.0072.

(27) Nanny, M. A.; Ratasuk, N. Characterization and Comparison of Hydrophobic Neutral and Hydrophobic Acid Dissolved Organic Carbon Isolated from Three Municipal Landfill Leachates. *Water Research* **2002**, *36* (6), 1572–1584. https://doi.org/10.1016/S0043-1354(01)00359-1.

(28) Wershaw, R. L.; Pinckney, D. J.; Llaguno, E. C.; Vicente-Beckett, V. NMR Characterization of Humic Acid Fractions from Different Philippine Soils and Sediments. *Analytica Chimica Acta* **1990**, *232*, 31–42. https://doi.org/10.1016/S0003-2670(00)81223-4.

(29) Wilson, M. A.; Hatcher, P. G. Detection of Tannins in Modern and Fossil Barks and in Plant Residues by High-Resolution Solid-State 13C Nuclear Magnetic Resonance. *Organic Geochemistry* **1988**, *12* (6), 539–546. https://doi.org/10.1016/0146-6380(88)90145-3.

(30) Zhou, Z.; Hua, B.; Cao, X.; Yang, J.; Olk, D. C.; Deng, B.; Liu, F.; Li, R.; Mao, J. Chemical Composition of Dissolved Organic Matter from Various Sources as Characterized by Solid-State NMR. *Aquat Sci* **2015**, *77* (4), 595–607. https://doi.org/10.1007/s00027-015-0405-8.

(31) Averett, R. C.; Leenheer, J. A.; McKnight, D. M.; Thorn, K. A. *Humic Substances in the Suwannee River, Georgia: Interactions, Properties, and Proposed Structures*; US Government Printing Office, 1994.

(32) El Hajjouji, H.; Merlina, G.; Pinelli, E.; Winterton, P.; Revel, J.-C.; Hafidi, M. 13C NMR Study of the Effect of Aerobic Treatment of Olive Mill Wastewater (OMW) on Its Lipid-Free Content. *Journal of Hazardous Materials* **2008**, *154* (1), 927–932. https://doi.org/10.1016/j.jhazmat.2007.10.105.

(33) Navalon, S.; Alvaro, M.; Garcia, H. Analysis of Organic Compounds in an Urban Wastewater Treatment Plant Effluent. *Environmental Technology* **2011**, *32* (3), 295–306. https://doi.org/10.1080/09593330.2010.497501.

(34) Huo, S.; Xi, B.; Yu, H.; Fan, S.; Jing, S.; Liu, H. A Laboratory Simulation of in Situ Leachate Treatment in Semi-Aerobic Bioreactor Landfill. *Water SA* **2008**, *34* (1), 133–140.

(35) Marche, T.; Schnitzer, M.; Dinel, H.; Paré, T.; Champagne, P.; Schulten, H.-R.; Facey, G. Chemical Changes during Composting of a Paper Mill Sludge–Hardwood Sawdust Mixture. *Geoderma* **2003**, *116* (3), 345–356. https://doi.org/10.1016/S0016-7061(03)00108-3.

(36) Piterina, A. V.; Barlett, J.; Pembroke, J. T. 13C-NMR Assessment of the Pattern of Organic Matter Transformation during Domestic Wastewater Treatment by Autothermal Aerobic Digestion (ATAD). *International Journal of Environmental Research and Public Health* **2009**, *6* (8), 2288–2306. https://doi.org/10.3390/ijerph6082288.
(37) Villaescusa, I.; Fiol, N.; Cristiani, F.; Floris, C.; Lai, S.; Nurchi, V. M. Copper (II) and Nickel (II) Uptake from Aqueous Solutions by Cork Wastes: A NMR and Potentiometric Study. *Polyhedron* **2002**, *21* (14–15), 1363–1367.
(38) Lewis, R.; van Leeuwen, J. A.; Smernik, R. J.; Chow, C. W. K.; Everson, A.; Nothrop, S. C.; Beecham, S. Changes in the Organic Character of Post-Coagulated *Pinus Radiata* Sulfite Pulp Mill Wastewater under Aerated Stabilization Basin Treatment—A Laboratory Scale Study. *Chemical Engineering Journal* **2011**, *175*, 160–168. https://doi.org/10.1016/j.cej.2011.09.089.
(39) Mosse, K. P. M.; Patti, A. F.; Smernik, R. J.; Christen, E. W.; Cavagnaro, T. R. Physicochemical and Microbiological Effects of Long- and Short-Term Winery Wastewater Application to Soils. *Journal of Hazardous Materials* **2012**, *201–202*, 219–228. https://doi.org/10.1016/j.jhazmat.2011.11.071.
(40) Calderón, V.; García, F. C.; De La Peña, J. L.; Maya, E. M.; García, J. M. Synthesis and Characterization of New Aromatic Polyamides Bearing Crown Ethers or Their Dipodal Counterparts in the Pendant Structure. I. Benzo-12-Crown-4 and Ortho-Bis(2-Ethoxyethoxy)Benzene. *Journal of Polymer Science Part A: Polymer Chemistry* **2006**, *44* (7), 2270–2281. https://doi.org/10.1002/pola.21339.
(41) Gressel, N.; McGrath, A. E.; McColl, J. G.; Powers, R. F. Spectroscopy of Aqueous Extracts of Forest Litter. I: Suitability of Methods. *Soil Science Society of America Journal* **1995**, *59* (6), 1715–1723. https://doi.org/10.2136/sssaj1995.03615995005900060030x.
(42) Lenz, S.; Böhm, K.; Ottner, R.; Huber-Humer, M. Determination of Leachate Compounds Relevant for Landfill Aftercare Using FT-IR Spectroscopy. *Waste Management* **2016**, *55*, 321–329.
(43) Barth, A. Fine-Structure Enhancement — Assessment of a Simple Method to Resolve Overlapping Bands in Spectra. *Spectrochimica Acta Part A: Molecular and Biomolecular Spectroscopy* **2000**, *56* (6), 1223–1232. https://doi.org/10.1016/S1386-1425(00)00228-6.
(44) Movasaghi, Z.; Rehman, S.; ur Rehman, Dr. I. Fourier Transform Infrared (FTIR) Spectroscopy of Biological Tissues. *Applied Spectroscopy Reviews* **2008**, *43* (2), 134–179. https://doi.org/10.1080/05704920701829043.
(45) He, Z.; Ohno, T. Fourier Transform Infrared and Fluorescence Spectral Features of Organic Matter in Conventional and Organic Dairy Manure. *Journal of Environmental Quality* **2012**, *41* (3), 911–919. https://doi.org/10.2134/jeq2011.0226.
(46) Socrates, G. *Infrared and Raman Characteristic Group Frequencies: Tables and Charts*; John Wiley & Sons, 2004.
(47) Soong, J. L.; Calderón, F. J.; Betzen, J.; Cotrufo, M. F. Quantification and FTIR Characterization of Dissolved Organic Carbon and Total Dissolved Nitrogen Leached from Litter: A Comparison of Methods across Litter Types. *Plant Soil* **2014**, *385* (1), 125–137. https://doi.org/10.1007/s11104-014-2232-4.
(48) Stewart, D. Fourier Transform Infrared Microspectroscopy of Plant Tissues. *Appl Spectrosc* **1996**, *50* (3), 357–365. https://doi.org/10.1366/0003702963906384.

(49) Bolyard, S. C.; Reinhart, D. R.; Richardson, D. Conventional and Fourier Transform Infrared Characterization of Waste and Leachate during Municipal Solid Waste Stabilization. *Chemosphere* **2019**, *227*, 34–42. https://doi.org/10.1016/j.chemosphere.2019.04.035.

(50) Coates, J. Interpretation of Infrared Spectra, a Practical Approach. *Encyclopedia of analytical chemistry* **2000**, *12*, 10815–10837.
